# Predictive modeling of single-cell DNA methylome data enhances integration with transcriptome data

**DOI:** 10.1101/gr.267047.120

**Published:** 2021-01

**Authors:** Yasin Uzun, Hao Wu, Kai Tan

**Affiliations:** 1 Center for Childhood Cancer Research, The Children's Hospital of Philadelphia, Philadelphia, Pennsylvania 19104, USA;; 2 Department of Biomedical and Health Informatics, The Children's Hospital of Philadelphia, Philadelphia, Pennsylvania 19104, USA;; 3 Department of Genetics, University of Pennsylvania, Philadelphia, Pennsylvania 19104, USA;; 4 Penn Epigenetics Institute, University of Pennsylvania, Philadelphia, Pennsylvania 19104, USA;; 5 Department of Pediatrics, University of Pennsylvania, Philadelphia, Pennsylvania 19104, USA

## Abstract

Single-cell DNA methylation data has become increasingly abundant and has uncovered many genes with a positive correlation between expression and promoter methylation, challenging the common dogma based on bulk data. However, computational tools for analyzing single-cell methylome data are lagging far behind. A number of tasks, including cell type calling and integration with transcriptome data, requires the construction of a robust gene activity matrix as the prerequisite but challenging task. The advent of multi-omics data enables measurement of both DNA methylation and gene expression for the same single cells. Although such data is rather sparse, they are sufficient to train supervised models that capture the complex relationship between DNA methylation and gene expression and predict gene activities at single-cell level. Here, we present methylome association by predictive linkage to expression (MAPLE), a computational framework that learns the association between DNA methylation and expression using both gene- and cell-dependent statistical features. Using multiple data sets generated with different experimental protocols, we show that using predicted gene activity values significantly improves several analysis tasks, including clustering, cell type identification, and integration with transcriptome data. Application of MAPLE revealed several interesting biological insights into the relationship between methylation and gene expression, including asymmetric importance of methylation signals around transcription start site for predicting gene expression, and increased predictive power of methylation signals in promoters located outside CpG islands and shores. With the rapid accumulation of single-cell epigenomics data, MAPLE provides a general framework for integrating such data with transcriptome data.

Recent advances in single-cell genomics provide the opportunity to capture a variety of epigenomic signatures at single-cell resolution, including histone modification (Grosselin et al. 2019), DNA methylation (Smallwood et al. 2014; Urich et al. 2015; Luo et al. 2017, 2018; Mulqueen et al. 2018), chromatin accessibility (Buenrostro et al. 2015; Chen et al. 2018), and long-range chromatin interaction (Nagano et al. 2013; Stevens et al. 2017). In particular, single-cell DNA methylome analysis can provide quantitative and high-resolution measurement of cell type–specific epigenomic landscape in both development and disease, because the mammalian embryonic development is associated with dynamic changes in DNA methylation at *cis*-regulatory elements and genome-wide deregulation of DNA methylation is associated with many types of cancer (Greenberg and Bourc'his 2019).

Identifying genome-wide methylation signatures at single-cell resolution with an unbiased technique such as bisulfite sequencing (BS-seq) comes with unique challenges. Technically, the data are sparse and genomic coverage is rather limited (∼5% of the genome per cell on average), even for deeply sequenced samples with more than 5 million reads per cell (Luo et al. 2017). Biologically, the interpretation of methylome signal is context dependent. Whole-genome DNA methylome data suggest that gene body methylation is positively correlated with gene expression in embryonic stem cells (Lister et al. 2009; Greenberg and Bourc'his 2019); in other cell types such as post-mitotic neurons, genic methylation is negatively correlated with gene expression (Lister et al. 2013; Lee et al. 2019). These observations indicate that the regulatory roles of DNA methylation are both genomic feature– and cell type–specific. Single-cell multi-omics studies suggest significant correlation (both positive and negative) between expression and gene body methylation for only a limited number of genes (Hu et al. 2016; Angermueller et al. 2016a). Additionally, mean promoter methylation is significantly negatively correlated with gene expression only for a fraction of promoters in individual cells (Angermueller et al. 2016a; Clark et al. 2018; Argelaguet et al. 2019).

The lack of well-defined association between DNA methylation and gene expression poses obstacles for the analysis and integration of this type of epigenomic data. Identifying cell types is relatively straightforward in scRNA-seq data using clustering techniques and marker genes. However, the same approach does not work well for single-cell DNA methylation data because of the lack of clear association between methylation and gene expression. Consequently, it is difficult to use marker genes to assign cell types in this case. In terms of data integration, several computational methods have been developed for integrating different types of single-cell data (Korsunsky et al. 2019; Stuart et al. 2019; Welch et al. 2019; Tran et al. 2020). However, without accurate input representing gene activities in single-cell methylation data, these methods cannot achieve reliable integration except for certain cell types, such as neurons, which have strong methylation signals in the gene bodies. Therefore, a robust estimate of methylation-based gene activity score remains a bottleneck for accurate integration of DNA methylation data with other types of single-cell omics data. Resolving this bottleneck can pave the way for comparative analysis of gene regulation mechanisms across cell populations in a complex tissue.

Recently, true multi-omics protocols (i.e., joint profiling of transcriptome and DNA methylome of the same cells) have started to emerge. It has already been shown that several hundreds of cells can be sequenced for gene expression and DNA methylation in the same cell in a single experiment (Angermueller et al. 2016a; Clark et al. 2018). Here, we aim to take advantage of such data and develop a supervised learning framework, called methylome association by predictive linkage to expression (MAPLE), for predicting gene activity score based on single-cell DNA methylation data. To this end, we aim to develop gene-dependent and cell-dependent statistical features as the input to an ensemble learning framework.

## Results

### Overview of the method

We independently verified the findings related to association between DNA methylation and gene expression by computing the correlation between the expression and promoter and gene body methylation in single-cell multi-omics data sets. As a result, we found that for only a small fraction of the promoters, the negative correlation between the methylation and the gene expression was significant. In addition, methylation of many promoters are positively correlated (Supplemental Fig. S1; Supplemental Tables S1, S2). Similarly, many more genes have negative correlation between body methylation and gene expression than those with positive correlation. Taken together, both published results and our own analysis suggest that there is not a straightforward approach for inferring gene activity levels using single-cell DNA methylation data sets. Instead, the correlation between the gene expression and methylation is gene dependent.

We hypothesize that the common patterns of promoter methylation-gene expression association for groups of genes can be modeled by using a supervised learning model. We developed two classes of statistical features as input to the supervised predictor. Promoters overlapping with CpG islands or shores have distinctive response to methylation (Weber et al. 2007; Deaton and Bird 2011; Jones 2012; Greenberg and Bourc'his 2019), and overall promoter CpG frequency has been used as a feature for predicting gene expression using bulk DNA methylation data (Kapourani and Sanguinetti 2016). In MAPLE, we used CpG frequency at higher resolution, as a vector of CpG frequencies of multiple genomic bins tiled across the promoter. This step generates the gene-dependent but cell-independent feature set to be used in the learning model. For the gene- and cell-dependent feature set, we computed the methylation level of each promoter bin for all cells and genes.

**Figure 1.**
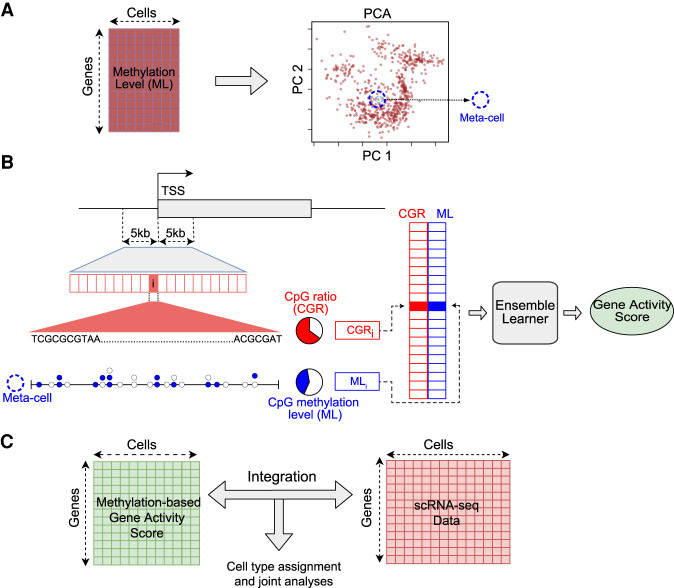
Schematic overview of the computational framework. (*A*) Generation of meta-cells from single-cell DNA methylation data. The gene-by-cell DNA methylation matrix is used for principal component analysis (PCA) to reduce the dimensionality. Each point in the PCA plot is a single cell. A meta-cell is the set of *k*-nearest cells to an individual cell in the PCA space. (*B*) Prediction of gene activity level by combining DNA methylation and sequence information in promoter. (TSS) Transcription start site; (ML) methylation level. Each horizontal box represents a genomic bin. Each circle represents a CpG site; a filled circle represents methylated cytosine, and an empty circle represents unmethylated cytosine in a meta-cell. CpG ratio is defined as the percentage of CpG dinucleotides in a genomic bin. CpG methylation level is defined as the ratio of the number of methylated CpG calls to all CpG calls. (*C*) Integration of single-cell methylation and single-cell RNA-seq data based on predicted gene activity scores using single-cell DNA methylation data.

The sparsity of single-cell bisulfite sequencing data poses a significant challenge for machine learning approaches. Many promoter regions have a limited number of CpG sites covered in each single cell, and dividing the promoter region into multiple bins further exacerbates the sparsity problem, resulting in either bins with no overlapping calls at all, or insufficient calls to make a reliable estimation of the methylation level of the bins. To alleviate this problem, we resorted to the concept of “meta-cell,” essentially borrowing information from neighboring single cells. Such an approach has been used to analyze other types of single-cell data (Wagner et al. 2017; Gong et al. 2018; van Dijk et al. 2018; Zhu et al. 2020). Each meta-cell represents a group of individual cells that are in a similar state with a specific cell in the data (Fig. 1A; Methods). Although this approach results in a slight loss of data resolution, it provides a reliable estimation of CpG level for the vast majority of meta-cells, even with small neighborhood sizes (Supplemental Fig. S2). Moreover, each single cell has its own unique neighborhood; thus, no two meta-cells are expected to be identical, and the single-cell nature of the data is preserved.

Once the meta-cells are identified, the CpG methylation level for each meta-cell is computed for 500-bp bins across the promoter region, resulting in 20 features for each gene and each meta-cell. Together with the CpG frequencies of these bins, they constitute the two classes of features for each gene-meta-cell pair (Fig. 1B; Methods). These features are used as the input to the supervised learning model to infer the gene activity level in the meta-cell. For downstream analysis, a DNA methylation–based gene activity matrix is constructed, and a desired method can be used to integrate the methylome data with gene expression data, for reliable assignment of cell types in the data and other joint multi-omics analyses (Fig. 1C).

Many supervised learning methods can be used to predict the gene activity level of the cells. We selected one commonly used, representative predictor from three broad categories: artificial neural networks, regression-based models, and decision tree–based models. Convolutional neural networks (CNNs) is a class of deep-learning neural networks, which have gained popularity in image processing and bioinformatics (Alipanahi et al. 2015; Angermueller et al. 2016b, 2017). Our CNN architecture resembles the method described in Singh et al. (2016) for predicting gene expression using bulk histone mark ChIP-seq data. However, instead of multiple histone marks, we used two classes of features, namely, CpG dinucleotide frequencies and CpG methylation levels for genomic bins surrounding the TSS (Fig. 1B; Methods). Elastic net (EN) is a regularized regression method that combines the least absolute shrinkage and selection operator (LASSO) and ridge regression models. Random forest (RF) consists of multiple decision trees, each of which is trained with random subsamplings from the training data, and the result is obtained by combining the outputs of all decision trees in a democratized manner. As the baseline method, we computed the mean promoter demethylation level (MPD) (ratio of unmethylated CpGs to all CpG calls in the promoter) as a predictor of gene activity level.

### Supervised learning improves accuracy of gene activity prediction

We benchmarked the performance of MAPLE using four published single-cell multi-omics data sets generated using two different protocols (Supplemental Table S1). The data sets of Angermueller and colleagues (Angermueller et al. 2016a) and Hernando-Herraez and colleagues (Hernando-Herraez et al. 2019) were generated using single-cell genome-wide methylome and transcriptome sequencing (scM&T-seq), and the data sets of Clark et al. (2018) and Argelaguet and colleagues (Argelaguet et al. 2019) were generated using single-cell nucleosome, methylation and transcription sequencing (scNMT-seq). For all data sets, the transcriptome and DNA methylome were jointly profiled for the same single cells. To evaluate the overall performance of the ensemble approach, we performed both internal and external cross validation, that is, training a predictor with one data set and predicting the gene activity levels using the remaining three data sets.

We evaluated the prediction accuracy using two metrics, Spearman's correlation and median squared error. We chose Spearman's correlation because it can capture both linear and nonlinear relationships in the data. We observed that the performance of the individual learners was sensitive to the selection of the training and test set data, especially for CNN and RF (Supplemental Fig. S3). These two methods performed well on the data set of Angermueller et al. (2016a) based on internal cross validation, but poorly based on external cross validation, indicating overfitting. On the other hand, their performance was low based on internal cross validation using the data set of Argelaguet et al. (2019), but higher based on external cross validation using the same data set as the training set. This is possibly because the Argelaguet data set has the largest number of cells, which captured the variation in the data better and alleviated the overfitting to some extent. Hence, the performance of these two methods is dependent on the training set and not stable. Finally, the overall performance of EN was the lowest, possibly owing to the relative simplicity of the model, which is unable to capture the complexity in the data. Therefore, to achieve a consistently improved performance independent of training and test data, we applied an ensemble approach by combining multiple predictors (Mendes-Moreira et al. 2012).

There are different ways for combining component predictors to build an ensemble predictor, such as unweighted average, weighted average, and stacking (Methods). We evaluated all three approaches and found that they have similar performance across all combinations of training and test data sets (Supplemental Fig. S4). Therefore, we chose the unweighted average approach because of its simplicity.

**Figure 2.**
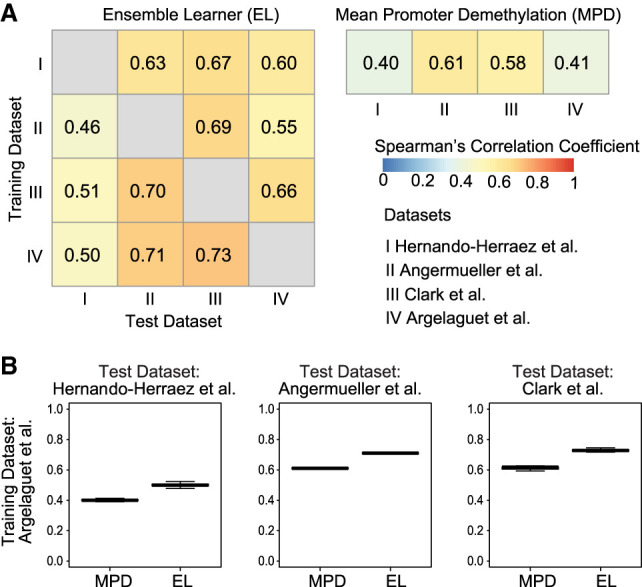
Prediction accuracy of gene expression using DNA methylation data and ensemble learning. (*A*) Heatmap showing global Spearman's correlation coefficients between observed gene expression and predicted gene activity for all genes across all cells in a data set. Rows represent training data sets, and columns represent test data sets. Row and column Roman numerals correspond to the data sets shown. (*B*) Distribution of Spearman's correlation coefficients across genes using the data set of Clark et al. (2018) as the training set. Each data point represents one cell. MAPLE (EL) correlations are significantly higher than those of MPD (*P* < 10^−16^, one-sided *t*-test for all three comparisons).

Ensemble-based MAPLE clearly outperformed MPD in terms of correlation for the internal cross-validation tests (Supplemental Fig. S5). Regarding cross-data set tests, across the 12 training-test data set pairs, MAPLE achieved an average global Spearman's correlation of 0.62 across all genes and cells (Fig. 2A). In comparison, the baseline predictor using MPD as the feature gave an average global Spearman's correlation of 0.5, which means MAPLE had 24% average performance gain over MPD (*P* = 0.003, one-sided *t*-test), reaching >60% improvement for certain data sets (Supplemental Table S3). The Spearman's correlation across genes is also higher for MAPLE compared to the baseline predictor (Fig. 2B; Supplemental Fig. S6). We observed the same trend when using a median squared error as an alternative metric (Supplemental Figs. S7, S8). Taken together, these results show that a supervised predictor using both CpG frequency and methylation level can substantially enhance the accuracy of predicting gene expression activity at the single-cell level.

Next, we investigated the predictive power of individual features by computing a feature importance score using the random forest model. We found that feature importance score distributions are similar across all four data sets (Supplemental Fig. S9). For both DNA sequence–based features (CpG ratio) and methylation rate features, the genomic bins that are closer to the TSS had higher importance. The importance score distribution of sequence-based features was symmetric around TSS, with the importance score attenuating after ±2 kb. In contrast, importance scores of the methylation rate features showed asymmetric distribution. Methylation rate of two bins immediately downstream from TSS had the highest predictive power, followed by the bin immediately preceding TSS. The importance score decreased after 1 kb for the upstream region and 2 kb for the downstream region.

### Predicted gene activity improves identification of cell types

Because one of the most important utilities of single-cell data is to identify different cell types/states in a heterogeneous population, we next evaluated our trained predictor on a single-cell DNA methylation data set containing 3377 mouse neurons that were sequenced with the single-nucleus methylcytosine sequencing (snmC-seq) protocol (Luo et al. 2017). In this study, Luo et al. (2017) identified different neuronal subtypes using gene body non-CpG (mCH) methylation, which is known to be inversely correlated with the expression level in adult neurons (Mo et al. 2015).

**Figure 3.**
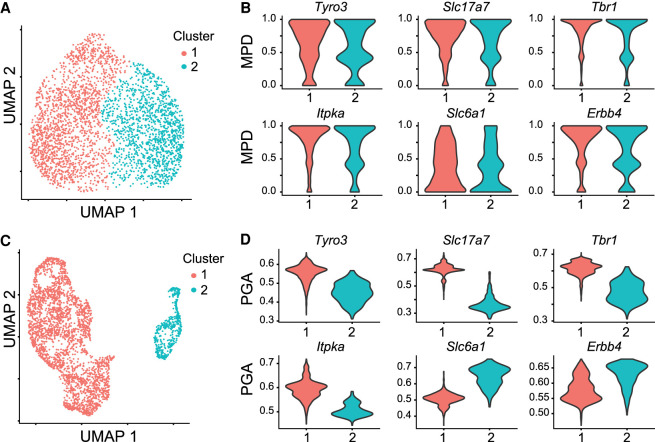
Predicted gene activity using methylome data improves cell subtype identification for neurons. (*A*) UMAP of clustering result generated using mean promoter demethylation (MPD) as the input. (*B*) Violin plot of MPD values for marker genes for excitatory (*Tyro3*, *Slc17a7*, *Tbr1*, *Itpka*) and inhibitory (*Slc6a1*, *Erbb4*) neurons. (*C*) Same as *A* but using predicted gene activity (PGA) as the input. (*D*) Same as *B* but using predicted gene activity as the input.

When we used the CpG (instead of non-CpG to be general) MPD values of all genes for dimensionality reduction, we observed no separation of cell types in the data, and the MPD values of the marker genes for the neuronal subtypes were indistinguishable across the cell populations (Fig. 3A,B; Supplemental Fig. S10). In contrast, when we clustered the cells using gene activity levels of all genes predicted by MAPLE (trained on data from Clark et al. [2018]), we identified two main clusters that were clearly separated (Fig. 3C). The larger cluster showed higher gene activity for excitatory neuron markers, such as *Tyro3, Slc17a7, Tbr1,* and *Itpka*, and the smaller cluster showed higher gene activity for inhibitory neuron markers, such as *Slc6a1* and *Erbb4* (Fig. 3D). The clustering result was consistent with that reported by Luo et al. (2017), with >99% of the cells in the excitatory neuron cluster and 95% of the cells in the inhibitory cluster matching those reported by the original study, respectively. We observed a similar trend in a larger set of excitatory and inhibitory marker genes (Supplemental Figs. S11–S14).

To further evaluate the utility of predicted gene activity for cell type identification, we applied MAPLE to identify cell clusters in the embryoid body (EB) population in the multi-omics data set by Clark et al. (2018) using data from Angermueller et al. (2016a) as the training set. In this study, Clark et al. (2018) differentiated mouse embryonic stem cells to EB. Using expression data, they reported that the differentiated population clusters into two main clusters, one is labeled as pluripotent due to high expression of pluripotency genes and the other represents differentiated cells with the opposite pattern.

**Figure 4.**
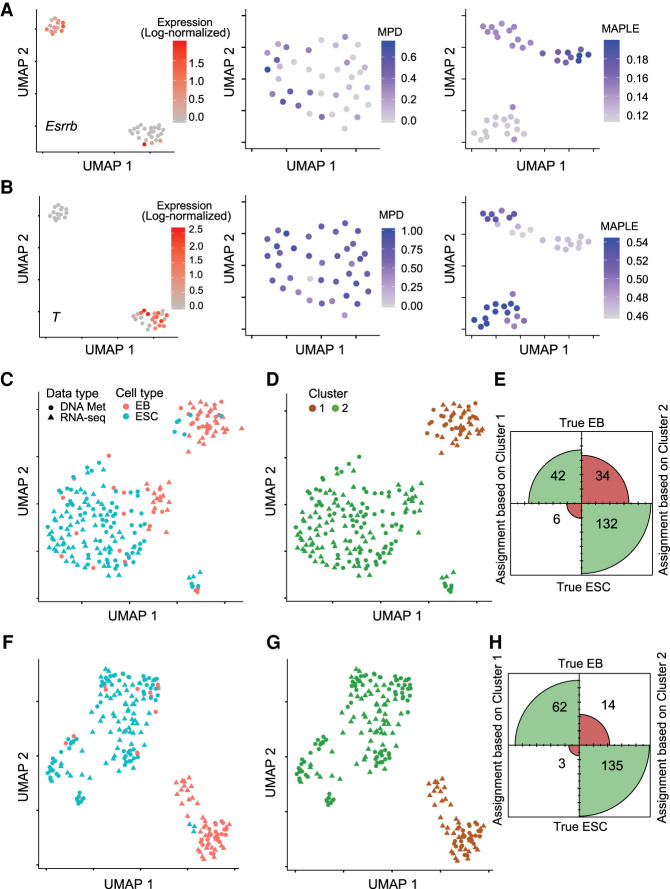
Predictive modeling improves integration with transcriptome data of cell lines. (*A*) Cell heterogeneity based on transcriptome and DNA methylome data. (*Left*) UMAP using RNA-seq data as the input. Color scale represents the log-normalized (using Seurat) expression level (read counts) of *Esrrb* for EBs. (*Middle*) UMAP using mean promoter demethylation as the input. Color scale represents the MPD (1 − mean methylation level) level of the *Esrrb gene.* (*Right*) UMAP using MAPLE-predicted gene activity based on DNA methylation data as the input. Color scale represents the MAPLE-predicted gene activity levels of *Esrrb*. (*B*) Same as *A*, but for the *T* gene. (*C*) UMAP based on integrated RNA-seq and DNA methylation data. Mean promoter demethylation (MPD) was used as the input for data integration using Seurat. (EB) embryoid body; (ESC) embryonic stem cell. (*D*) Density clustering of the data shown in the UMAP in *C*. (*E*) Confusion matrix plot based on the clustering result shown in *D*, illustrating the agreement between cell type assignment based on clustering and true cell type. Size of each quadrant is proportional to the number of cells classified. (*F*) Same as *C*, but using predicted gene activity as the input. (*G*) Same as *D*, but using predicted gene activity as the input. (*H*) Same as *E*, but using predicted gene activity as the input. χ^2^ test *P*-value for the confusion matrices in *G* and *H* is 0.002.

Using the MPD levels of promoters as the input for clustering did not recapitulate the heterogeneity observed using the transcriptome data. On the other hand, the same analysis using MAPLE-predicted activities of all genes as the input resulted in two clusters that have similar sizes to the clusters defined using transcriptome data alone. Moreover, *Esrrb*, a pluripotency marker gene showed high levels of activity in one cluster, whereas transcription factor *T*, a differentiation marker, displayed the opposite pattern (Fig. 4A,B), consistent with the pattern of heterogeneity based on the transcriptome data. Similar separation was evident for many additional marker genes for the two cell types (Supplemental Figs. S15, S16). To test the reliability of the separation of the cell types in the MAPLE input, we computed the dimensionality reduction with different parameter settings, and we observed that the separation was evident across all parameter settings (Supplemental Figs. S17, S18).

In summary, these results show that use of a predictive modeling approach can significantly improve our ability to identify cell types and reveal heterogeneity in single-cell methylation data. Notably, even in this case of extremely small number of cells, using the meta-cell approach to predict gene activity did not lead to loss of resolution that could impede analysis of heterogeneity.

### Predicted gene activity enhances integration with scRNA-seq data

We reasoned that accurate prediction of gene activity level can significantly enhance the integration of single-cell methylome data with transcriptome data. To test this hypothesis, we took advantage of published true multi-omics data sets in which DNA methylome and transcriptome were measured for the same single cells. Therefore, we know the ground truth about the matching of the two data types for a given cell. We first used the data set by Clark et al. (2018) that is composed of differentiated embryoid body cells and undifferentiated embryonic stem cells (ESCs). Using predicted gene activity levels by either MAPLE or the MPD method, we integrated the methylome and transcriptome data using Seurat (Stuart et al. 2019). We then clustered the cells using the coembedded transcriptome and methylome data produced by Seurat. We computed the fraction of cells in the methylome data that were assigned to the correct cluster, using a *k*-nearest neighbor (*k*-NN) classifier and cells from single-cell RNA data. In contrast to MPD, clusters computed based on MAPLE-predicted gene activity showed higher homogeneity (i.e., greater fraction of the cells in each cluster belongs to the same type) (Fig. 4C–H; Supplemental Fig. S19), suggesting improved accuracy in data integration.

**Figure 5.**
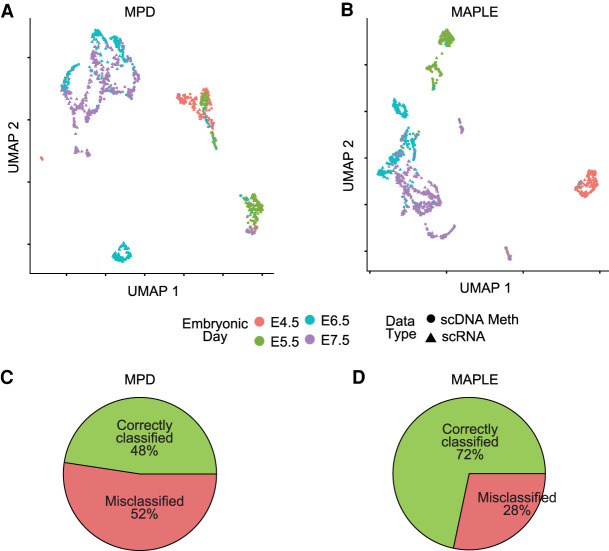
Predictive modeling improves integration with transcriptome data of primary tissues. (*A*) UMAP plots for integrated expression and DNA methylation data. Mean promoter demethylation (MPD) was used as the input for data integration using Seurat. (*B*) Same as *A*, but using MAPLE-predicted gene activity as the input. (*C*) Pie chart showing the percentage of correctly and misclassified cells using scDNA-methylation data, based on *k*-nearest neighbor (*k*-NN) classification on the scRNA-seq cells for the MPD-based UMAP in *A*. (*D*) Same as *C*, but for the MAPLE-based UMAP in *B*. χ^2^ test *P*-value for the comparison between correct and misclassifications in *C* and *D* is 3.6 × 10^−10^.

We further evaluated the performance of data integration using a larger data set on primary tissue. The data set includes 850 cells from four different embryonic time points (Argelaguet et al. 2019) during mouse gastrulation. Each cell was sequenced with multiple modalities, including chromatin accessibility, DNA methylation, and RNA expression. We integrated the single-cell methylome data with the expression data, as described above, and computed the fraction of cells in the methylome data that were assigned to the correct cluster. Using gene activity predicted by MPD, neither linear (PCA) nor nonlinear (UMAP) dimensionality reduction methods on the integrated data resulted in accurate matching of cells from the same developmental stage (Fig. 5A,B; Supplemental Fig. S20). In comparison, using MAPLE data as the input for integration, the resulting integrated data showed much higher fractions of matched cells based on the two data types for all four developmental stages (Fig. 5C,D; Supplemental Figs. S20, S21). Although part of the populations from E6.5 and E7.5 has some overlap in the integrated data based on MAPLE input, this outcome results from the biological nature of the data, as it is observed in all data modalities including gene expression (Argelaguet et al. 2019).

## Discussion

Although recent advances in single-cell DNA methylome sequencing technologies improve our ability to study epigenetic heterogeneity, data analysis poses unique challenges, especially the issue of connecting the methylome and transcriptome data. Here, we addressed this challenge by developing a supervised learning approach to inferring gene activity based on DNA methylation data. The inferred gene activity score acts as an intermediate input for integrating the two types of data. A similar approach is commonly used to integrate single-cell ATAC-seq data with transcriptome data in which summed chromatin accessibility signal in the promoter and gene body is used as a proxy to gene activity and intermediate input for data integration. However, simply using summed DNA methylation signal in the promoter region does not work, as we showed using the mean promoter demethylation method, owing to the more complex relationship between DNA methylation and gene expression. Instead, we showed that predictive modeling better captures the relationship between DNA methylation and gene expression. As a result, the predicted gene activity score helps improve cell type identification in the methylome data as well as integration with transcriptome data.

For constructing meta-cells, we used the PCA that is computed with methylation levels of the variably methylated promoters (VMPs) as the input. It might be argued that the presence of CpG-poor promoters can affect quality of constructed meta-cells. We evaluated this issue by computing the overlap between the VMPs and CpG-poor promoters (10% of all promoters having the lowest number of CpG sites). We found that a very small fraction of VMPs are CpG-poor (0.6%) and their removal has little effect (7.5%) on the adjacency matrix (Supplemental Table S4), demonstrating reliability of meta-cells.

Despite the fact that using information from neighboring cells may lead to minor loss of resolution, the reliability gained by using meta-cell for predicting gene activity outweighs its cost. The ability of MAPLE to identify subpopulations with a very small number of EB cells (Clark et al. 2018) shows that the use of meta-cells does not significantly impede the ability of our method for detecting heterogeneity.

Although MAPLE consistently outperforms MPD for all data sets we studied, the performance gain varied across the training and test data sets used. It has been reported that expression of genes having CpG island promoters have low sensitivity to DNA methylation change (Antequera 2003; Fan and Zhang 2009). We therefore investigated whether different gene sets contributed to the performance differences by MAPLE by categorizing genes into four groups: genes having promoters overlapping with CpG islands, CpG shores, CpG shelves, and open sea (all remaining genes). We found the performance of MAPLE is highest using genes in the open sea and lowest using genes with CpG islands (Supplemental Fig. S22). This result suggests that the expression of genes located in CpG islands, shores, and shelves has low sensitivity to changes in methylation signal. In contrast, genes in the open sea have a more dynamic expression pattern in response to methylation change.

Our analysis revealed some interesting biological insights. First, for many genes, promoter methylation and gene expression are positively correlated, which is inconsistent with the observation based on bulk data. This result suggests a more nuanced relationship between gene expression and promoter methylation that is dependent on cell type or state and not previously captured by bulk data. Second, we found that the importance of CpG methylation in predicting gene expression is not symmetric around TSS. Overall, the downstream region has higher predictive power than the upstream region. Furthermore, CpG methylation of the 500-bp region immediately downstream of the TSS has the greatest predictive power.

In our framework, we used promoter methylation because it can be directly associated with genes. It has been reported that gene-distal transcriptional enhancers also have unique DNA methylation signature (Lister et al. 2009; Stadler et al. 2011; Hon et al. 2013). We anticipate that inclusion of enhancer DNA methylation signals can further improve the accuracy of the predictive model. To this end, a major challenge is associating distal enhancers with their target genes. This question has been addressed extensively for bulk data using both experimental and computational approaches (He et al. 2014; Javierre et al. 2016; Cao et al. 2017; Jung et al. 2019). However, owing to unique challenges in single-cell data, additional research is warranted to address this important question. Finally, we used an ensemble learning approach in our method. Although we chose three representative predictors as components of the ensemble predictor, benefits of adding more predictors can be explored in future work.

## Methods

### Processing of public data sets

Data sets used in this study are listed in Supplemental Table S1. Briefly, we used four single-cell multi-omics data sets generated by two different experimental protocols, scM&T-seq (Angermueller et al. 2016a) and scNMT-seq (Clark et al. 2018). In addition, we used the snmC-seq (Luo et al. 2017) data set as the methylation-only data. We used Bismark methylation call files (COV files) when available from the authors or converted the methylation calls into Bismark COV format (Krueger and Andrews 2011). For each data set, we only used cells for which both scRNA-seq and sc-Methylome data were available.

For the single-cell RNA-seq data, we normalized the number of reads by the total number of reads per cell and obtained counts per million (CPM). CPM values were log transformed and were further normalized by the maximum log expression value in the data set to fit all data sets into the same range and make training and test sets compatible.

### Computing meta-cells

To alleviate the problem of data sparsity, we combined DNA methylation data from neighboring cells into meta-cells as follows. We first counted the CpG methylation calls in the ±5 kbp region flanking the transcription start site (TSS) of the genes and computed the methylation level as the ratio of methylated CpGs to all CpG calls in those regions. For regions with no cytosine calls for a particular cell, we used the mean methylation level across all promoters of that cell. Next, we performed principal component analysis on this methylation level matrix using the top 5000 promoters with highest variance of methylation across the cells. We then used the top *d* = 10 (*d* is an adjustable parameter in our method) principal components as the feature space to compute the Euclidean distance between each cell pair, because the total variance explained after the 10th principle component was minimal (Supplemental Fig. S23). Based on the distance, we defined the local neighborhood of each cell as the *k-*nearest cells. Each meta-cell corresponds to an actual cell in the data set, that is, the number of meta-cells is the same as the number of single cells in the data. Because we observed that there is minimal improvement in terms of non-empty bins for *k* larger than 20 (Supplemental Fig. S24) for all data sets in this study, we used *k* = 20.

We evaluated the effect of CpG-poor promoters for meta-cell as follows. First, we determined the 20 nearest neighbors of each cell using the top 10 dimensions of PCA and built an adjacency matrix. Next, we repeated this procedure when CpG-poor promoters are removed from the input gene set when computing the PCA. Then, we determined the number of changes in the two matrices with and without the CpG-poor promoters. Finally, we calculated the ratio of the number of changed edges to the total number of edges in the original adjacency matrix (with CpG-poor promoters) as the percentage of adjacency difference.

### Calculation of DNA methylation rate

Transcription start sites (TSSs) were defined based on the GENCODE annotation (release vm23) for mouse genome (release GRCm38). “Promoter” was defined as the region spanning 5 kbp upstream of and downstream from the TSS.

To compute CpG methylation rate, we first divided each promoter into 20 bins of 500-bp length. For each meta-cell, the numbers of methylated and unmethylated CpG sites for each bin were counted. The methylation rate of each bin was calculated by dividing the total number of methylated cytosines to all cytosines in that bin, considering all the cytosine calls for all cells in the corresponding meta-cell.

Selection of the bin size may have an effect on the performance. Very large bin sizes can lead to loss of resolution, whereas too small bin sizes will result in many bins with few CpG calls, leading to inaccurate estimates of methylation rates. To evaluate this issue, we performed external cross validation using different bin sizes. We found that there is a minor difference in performance using different bin sizes (Supplemental Fig. S25).

### Feature set

Two classes of features were computed: gene-dependent and cell-independent feature, and cell- and gene-dependent feature. For each gene, we extracted the sequence information for the ±5 kbp region around the TSS. For each 500-bp bin, we computed the CpG frequency, considering both strands, which resulted in 20 gene-dependent, cell-independent features. Cell- and gene-dependent features are CpG methylation rates of the 20 promoter bins of a gene in a given cell. They were calculated by using all methylation calls for all cells in the corresponding meta-cell.

### Training and testing the predictor

We randomly subsampled 100,000 cell-gene pairs from each training data set to speed up the training process. Three supervised learners, convolutional neural network (CNN), elastic net (EN), and random forest (RF), were trained with each of the four multi-omics data sets. We evaluated the performance with different parameter settings for individual learners using fivefold internal cross validation (Supplemental Figs. S26–S28). To avoid bias caused by different experimental protocols and/or data sets, we trained and tested the predictors using external cross validation, that is, training and testing using different data sets.

We trained the CNN models with 50 filters using the ReLU activation function for hidden layers and linear activation for the output layer. Kernel size was set to 5, and max pooling was set to size of 4. Mean squared error (MSE) was used as the loss function, and patience for early stopping was set to 10 epochs. Models were regularized by setting the dropout rate to 0.2 to avoid overfitting (Srivastava et al. 2014). We used the R keras package for the implementation of the model (https://cran.r-project.org/web/packages/keras/).

We trained the EN predictor (Friedman et al. 2010) by setting the value of *alpha* to 0.5. The value of the λ was determined using 10-fold internal cross validation. We used the R glmnet package to train the predictor with cv.glmnet function (https://cran.r-project.org/web/packages/glmnet/index.html).

Random forest (RF) predictors were constructed with 500 trees, in which each tree was grown with random subsampling of training data using 80% of the training samples. We used the R randomForest package (https://cran.r-project.org/web/packages/randomForest/) to build the RF models.

There are different rules for building an ensemble from the individual predictors. The unweighted average approach used in this study is the mean of the outputs of the individual predictors and does not depend on any prior assumptions on the underlying models. As an alternative, weighted average prioritizes some of the predictors over others based on some prior information. We first calculated the median correlations between predicted gene activity and observed gene expression from the cross validation results and used them as weights for combining the outputs of the predictors. In other words, the predictor that was associated with higher correlation in the training data received higher weight. As another alternative, we used median accuracy (1 − error) as the weight for each predictor.

A more complicated combination rule is the stacked learning (stacking) approach, in which a second layer predictor is trained on the outputs of the first layer predictors (Wolpert 1992; Breiman 1996) using training data. We tested stacking approach with three different second level predictors (belonging to the same family of learners used for the first layer): elastic net regression, artificial neural network, and random forest. This approach gave mixed results on the different data sets, offsetting the advantage of stability of ensemble approach. As a result, we decided to use the unweighted averaging approach, which is the simplest ensemble model and does not rely on any prior information. We compared the correlations between MAPLE and MPD using *t*-test, and we replicated the correlation coefficients achieved using MPD, so that there are the same number (12) of values for comparison of two matrices.

### Feature importance

We computed feature importance scores with the random forest model using the “importance” function in R random forest package (https://cran.r-project.org/web/packages/randomForest/). We used the MSE difference as the importance measure. It was calculated by training a model using all features, then iteratively permuting the values for one feature at a time and computing the difference between overall MSE and MSE obtained with the permuted feature.

### Integration of methylome and transcriptome data using predicted gene activity

We integrated single-cell DNA methylation data with single-cell RNA sequencing data using Seurat version 3 (Stuart et al. 2019). Only cells that have both expression and methylation data were used for the integration. After normalization of both data types, the top 3000 integration features (genes) were selected using the “SelectIntegrationFeatures” function. Then the integration anchors (cells) were selected with the “FindIntegrationAnchors” function using the integration features. The k.filter (number of neighbors) was set to 100, and normalization was set to SCT (Hafemeister and Satija 2019). The two data types were integrated by using those anchors and the “IntegrateData” function. Finally, we ran PCA,UMAP, and clustering on the integrated (coembedded) data, including cells from both data types.

### Software availability

MAPLE software is implemented in R (R Core Team 2019) and is freely available under the MIT license. Source code is available as Supplemental Code and at GitHub (https://github.com/tanlabcode/MAPLE.1.0).

## Competing interest statement

The authors declare no competing interests.

## Supplementary Material

Supplemental Material
