# Supplementary material for: Predictive modeling of single-cell DNA methylome data enhances integration with transcriptome data
Source: Genome Res. 2021 Jan;31(1):101–9. doi: 10.1101/gr.267047.120 (PMC7849382; doi:10.1101/gr.267047.120)
Supplement: Supplemental Material [file supp_31_1_101__index.html]

Predictive modeling of single-cell DNA methylome data enhances integration with transcriptome data — Supplemental Material 

# Predictive modeling of single-cell DNA methylome data enhances integration with transcriptome data

## Supplemental Material

- Supplemental\_Code.zip
- Supplemental\_Material.pdf
