# Supplementary material for: Predictive modeling of single-cell DNA methylome data enhances integration with transcriptome data
Source: Genome Res. 2021 Jan;31(1):101–9. doi: 10.1101/gr.267047.120 (PMC7849382; doi:10.1101/gr.267047.120)
Supplement: Supplemental Material [file supp_gr.267047.120_Supplemental_Material.pdf]

SUPPLEMENTARY INFORMATION

Predictive modeling of single-cell DNA methylome data enhances integration with transcriptome data

Yasin Uzun[1,2], Hao Wu[3,5] and Kai Tan[1,2,3,4,5]

[1]Center for Childhood Cancer Research, The Children's Hospital of Philadelphia, Philadelphia, PA 19104, USA

[2] Department of Biomedical and Health Informatics, The Children's Hospital of Philadelphia, Philadelphia, PA 19104, USA

[3] Department of Genetics, University of Pennsylvania, Philadelphia, PA 19104, USA

[4] Department of Pediatrics, University of Pennsylvania, Philadelphia, PA 19104, USA

[5] Penn Epigenetics Institute, University of Pennsylvania, Philadelphia, PA 19104, USA

Correspondence: Kai Tan (tank1@email.chop.edu)

TABLE OF CONTENTS

I. SUPPLEMENTAL TABLES 4

Supplemental Table S1. Public single cell sequencing datasets that were used in this study. 4

Supplemental Table S2. Number of genes for which gene expression and DNA methylation have significant correlation (adjusted p-value < 0.01). 4

Supplemental Table S3. Percentage of performance gain by ensemble learner over mean promoter demethylation (MPD) across datasets. 5

Supplemental Table S4. The effect of genes with CpG-poor promoters on the quality of meta-cells. 5

II. SUPPLEMENTAL FIGURES 6

Supplemental Fig. S1. Scatter plots showing the correlation between the promoters and gene body methylation and gene expression. 6

Supplemental Fig. S2. Violin plots showing the effect of using data from meta-cells, instead of individual cells for computation. 7

Supplemental Fig. S3. Performance of individual learners for gene activity prediction. 8

Supplemental Fig. S4. Performance comparison of different ensemble learning combination rules. 9

Supplemental Fig. S5. Performance comparison between Mean Promoter De-methylation (MPD) and Ensemble Learner (EL) as the predictor of gene expression. 10

Supplemental Fig. S6. Evaluation of prediction accuracy using Spearman's correlation across genes 11

Supplemental Fig. S7. Evaluation of prediction accuracy using median squared error across cells. 12

Supplemental Fig. S8. Evaluation of prediction accuracy using median squared error across genes. 13

Supplemental Fig. S9. Evaluation of feature importance using Random Forest models. 14

Supplemental Fig. S10. Clustering of neuronal dataset in Fig. 3a using different sets of PCA dimensions with MPD as the input. 15

Supplemental Fig. S11. MPD distributions of marker genes for excitatory neurons. 16

Supplemental Fig. S13. MAPLE gene activity distributions of marker genes for excitatory neurons. 18

Supplemental Fig. S14. MAPLE predicted gene activity distributions of marker genes for inhibitory neurons. 19

Supplemental Fig. S15. MPD distributions of marker genes for pluripotency and differentiation (EB) in the dataset of Clark et al. 20

Supplemental Fig. S16. MAPLE predicted gene activity distributions of marker genes for pluripotency and differentiation (EB) in the dataset of Clark et al. 21

Supplemental Fig. S17. UMAP plots for embryoid bodies (EBs) for the dataset of Clark et al. using various parameter settings. 22

Supplemental Fig. S18. Violin plots showing the MAPLE predicted gene activities for the pluripotent marker gene Esrrb and the differentiation marker gene T with different parameter settings for embryoid bodies (EBs) for the dataset of Clark et al using various parameter settings. 23

Supplemental Fig. S19. Classification accuracy for cells in the methylome data based on the integration of the dataset of Clark and colleagues (Clark et al. 2018). 24

Supplemental Fig. S20. Predictive modeling improves integration with transcriptome data of primary tissues. 25

Supplemental Fig. S21. Percentage of correct classification for the cells in the methylome data based on the integration of the dataset of Argelaguet and colleagues (Argelaguet et al. 2019) using two different training datasets. 26

Supplemental Fig. S22. Heatmaps showing Spearman's correlation coefficients between gene expression and MAPLE predicted gene activity for different sets of genes. 27

Supplemental Fig. S23. Elbow plots showing the percentage of variance in the data explained by each principal component in the principal component analysis, sorted from highest to lowest. 28

Supplemental Fig. S24. Violin plots showing the distribution of the number of non-empty bins for individual cells and meta-cells of varying sizes. 29

Supplemental Fig. S25. Sensitivity of MAPLE for different bin sizes. 30

Supplemental Fig. S26. Box plots showing the parameter tuning results with 5-fold cross validation for convolutional neural network predictor. 31

Supplemental Fig. S27. Box plots showing the parameter tuning results with 5-folds cross validation for elastic net predictor. 32

Supplemental Fig. S28. Box plots showing the parameter tuning results with 5-folds cross validation for random forest predictor 33

IV. SUPPLEMENTAL REFERENCES 34

I. SUPPLEMENTAL TABLES

Supplemental Table S1. Public single cell sequencing datasets that were used in this study.

Accession ID	Experimental Protocol	Data modalities	Pubmed ID (Reference)
GSE74535	scM&T-seq	Methylome and transcriptome	26752769 (Angermueller et al. 2016)
GSE121436	scM&T-seq	Methylome and transcriptome	31554804 (Hernando-Herraez et al. 2019)
GSE109262	scNMT-seq	Methylome, transcriptome and nucleosome occupancy	29472610 (Clark et al. 2018)
GSE121708	scNMT-seq	Methylome, transcriptome and nucleosome occupancy	31827285 (Argelaguet et al. 2019)
GSE97179	snmC-seq	Methylome	28798132 (Luo et al, Science, 2017)

Supplemental Table S2. Number of genes for which gene expression and DNA methylation have significant correlation (adjusted p-value < 0.01).
Datasets: I:Hernando-Herraez et al. II: Angermueller et al. III: Clark et al. IV: Argelaguet et al. TSS, transcription start site; N, negative correlation; P, positive correlation.

Dataset	TSS+/-5kb		TSS+/-2kb		Gene Body	
	N	P	N	P	N	P
I	1	13	10	59	2	2
II	20	10	13	43	12	2
III	31	36	22	44	44	33
IV	790	121	408	58	1696	397

Supplemental Table S3. Percentage of performance gain by ensemble learner over mean promoter demethylation (MPD) across datasets.
Performance was measured by correlation of predicted gene activity and observed expression. Datasets: I:Hernando-Herraez et al. II: Angermueller et al. III: Clark et al. IV: Argelaguet et al.

		Test Dataset			
		I	II	III	IV
Training Dataset	I		4%	15%	47%
	II	16%		18%	34%
	III	26%	15%		62%
	IV	25%	16%	26%	
	AVERAGE	22%	12%	20%	48%

Supplemental Table S4. The effect of genes with CpG-poor promoters on the quality of meta-cells.

CpG-poor promoters were defined as those whose number of CpG sites are ranked in the lowest 10 percentile. A total of 5,000 variably methylated promoters (VMPs) were used to compute the PCA, which was then used to compute the adjacency matrices. Adjacency difference means the difference in the adjacency matrices generated with and without CpG-poor promoters. Total edges means the number of edges (non-zero values) in the adjacency matrix. Datasets: I:Hernando-Herraez et al. II: Angermueller et al. III: Clark et al. IV: Argelaguet et al.

Dataset	#VM CpG poor promoters	% VM CpG poor promoters	Adjacency difference	# Total edges	% Adjacency difference
I	9	0.18	212	2,625	8
II	13	0.26	52	1,281	4
III	88	1.76	234	2,247	10
IV	13	0.26	1,096	14,112	8
**AVERAGE**	**30.8**	**0.6**	**398.5**	**5,066.3**	**7.5**

II. SUPPLEMENTAL FIGURES

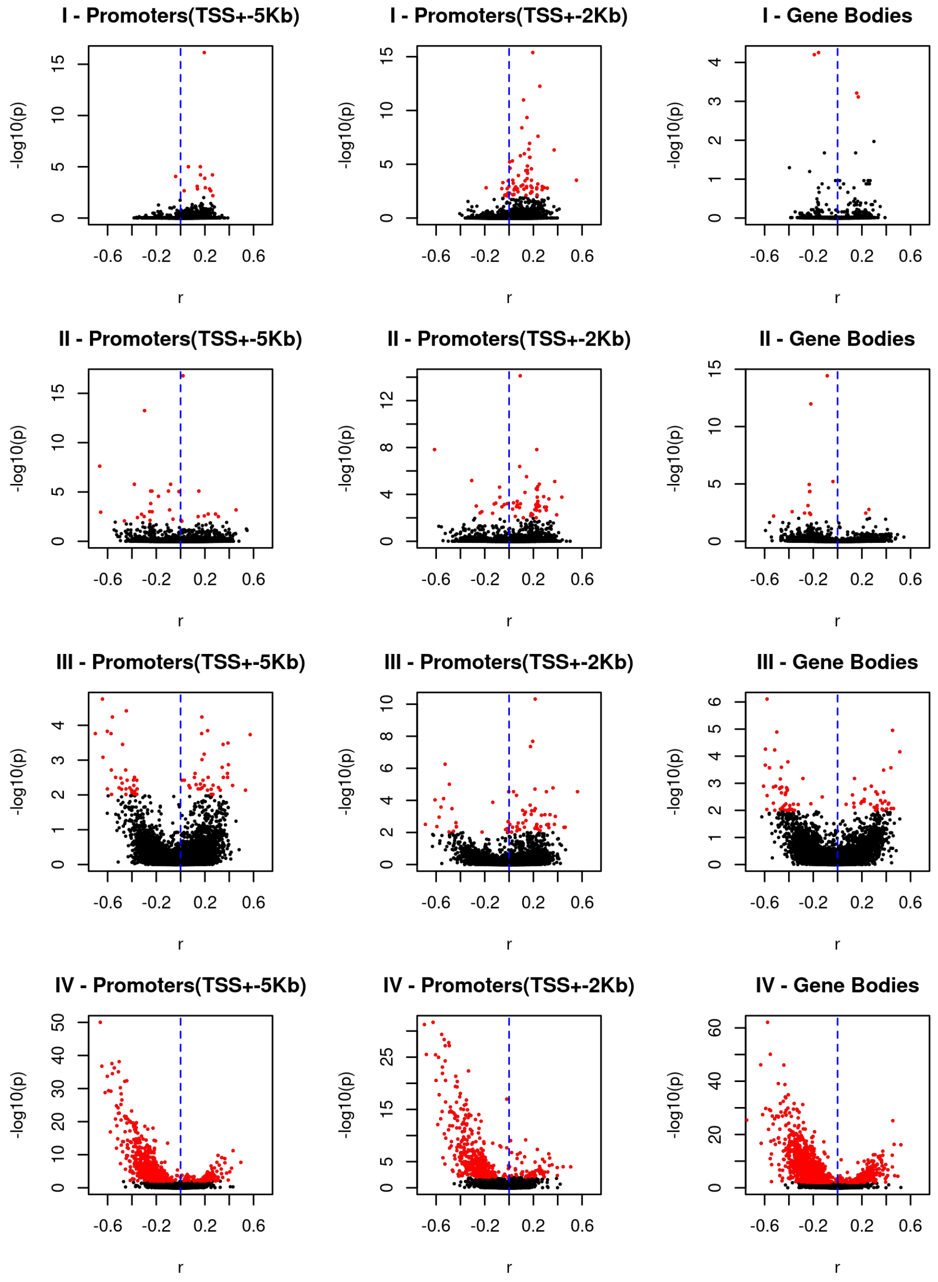

Supplemental Fig. S1. Scatter plots showing the correlation between the promoters and gene body methylation and gene expression.

Each row (I-IV) is a multi-omics dataset and each column is a region type (TSS +/-5kb, TSS +/-2kb, gene body). Datasets: I:Hernando-Herraez et al. II: Angermueller et al. III: Clark et al. IV: Argelaguet et al. For each plot, the X-axis shows the Spearman's correlation coefficient and the Y-axis shows the negative log 10 p-value (BH-corrected). Each point is a gene. Significant (adjusted p-value < 0.01) correlations are colored in red. Blue dashed line marks the correlation coefficient of 0.

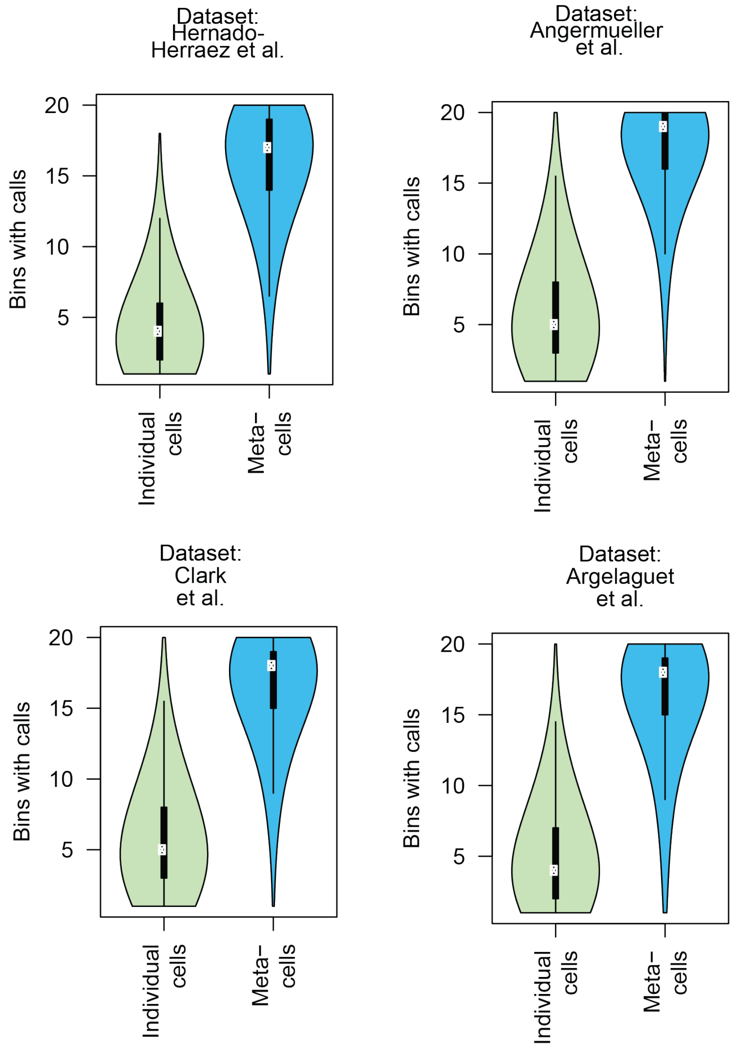

Supplemental Fig. S2. Violin plots showing the effect of using data from meta-cells, instead of individual cells for computation.

The +/-5kbp flanking region of TSS was divided into 500bp bins (20 bins). Each data point in plots is a meta-cell and gene pair and the Y-axis shows the number of bins having at least one overlapping cytosine call.

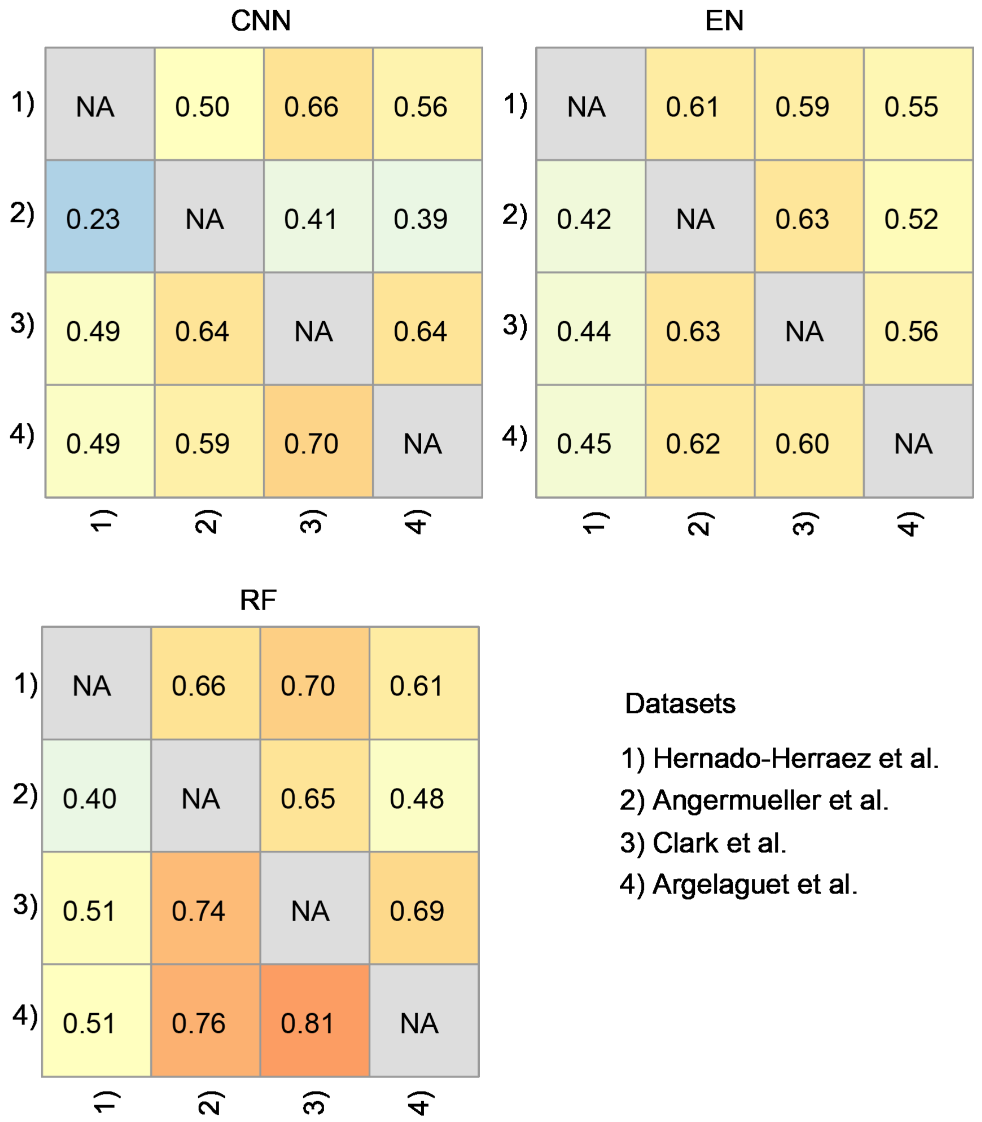

Supplemental Fig. S3. Performance of individual learners for gene activity prediction.

For each matrix, rows correspond to training sets and columns correspond to the test sets. The values in the cells are Spearman's correlation coefficients.

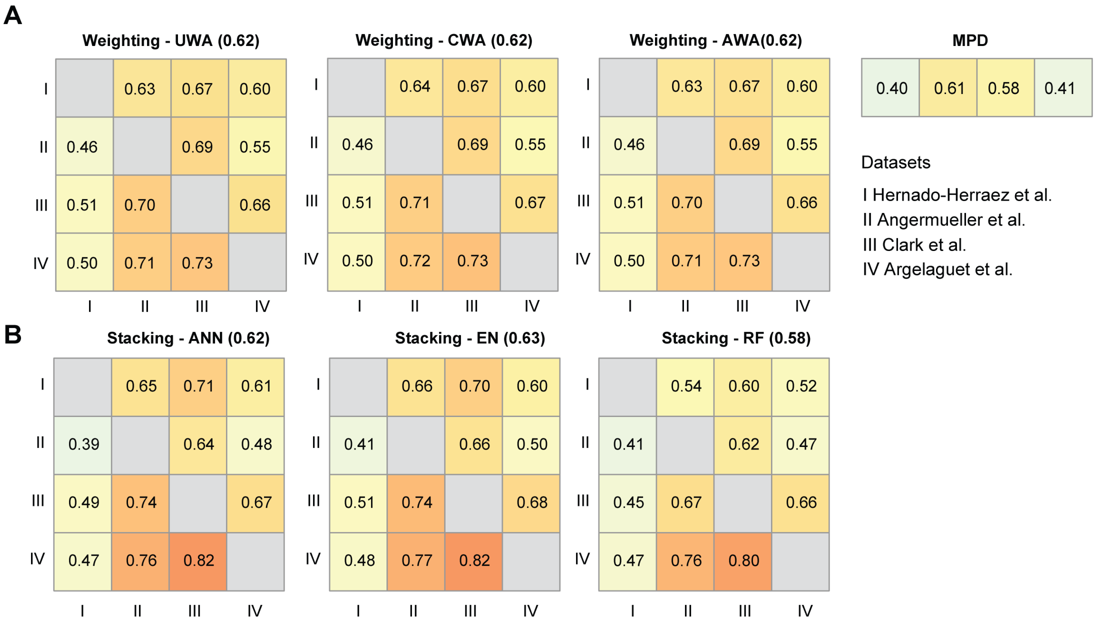

Supplemental Fig. S4. Performance comparison of different ensemble learning combination rules.

The rules of combining individual predictors were compared: unweighted (UWA), weighted averaging, and stacking. For weighted averaging, weights were determined either by Spearman's correlation coefficient computed with cross-validation (CWA) or by accuracy (1-error) computed with cross-validation (AWA). For stacking, the individual predictors were not given weights. Instead, they were combined into the ensemble by using a second-level predictor. ANN, Artificial Neural Network; EN, Elastic Net Regression; RF, Random Forest. MPD, Mean Promoter De-methylation. **A**) Performance of weighting rules. Heatmap showing global Spearman's correlation coefficients between observed gene expression and predicted gene activity for all genes across all cells in a dataset. Rows represent training datasets and columns represent test datasets. **B)** Performance of stacking rules.

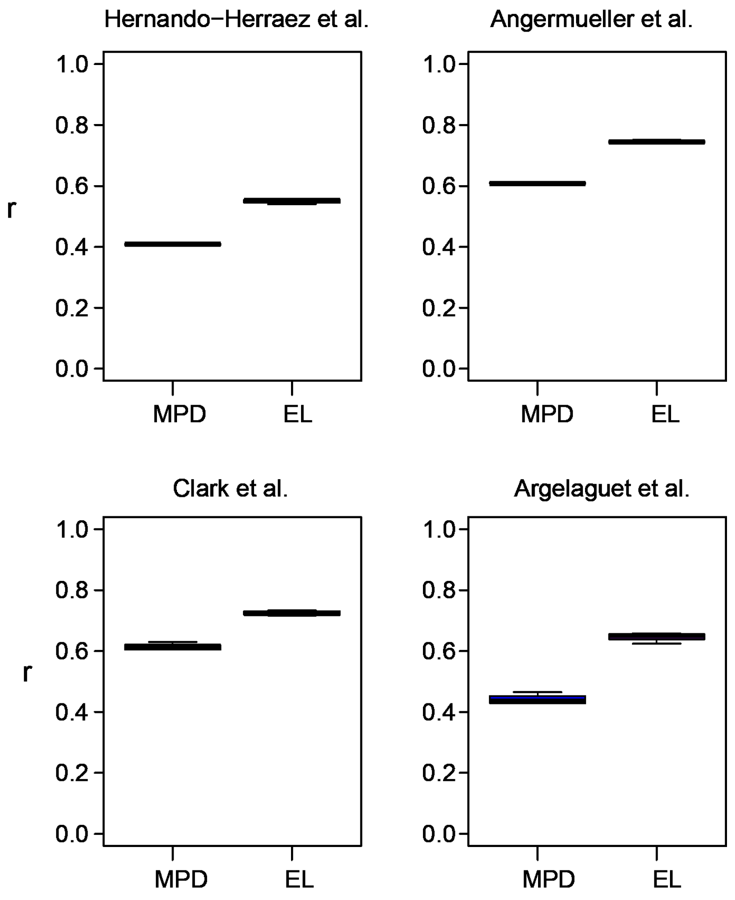

Supplemental Fig. S5. Performance comparison between Mean Promoter De-methylation (MPD) and Ensemble Learner (EL) as the predictor of gene expression. Each boxplot corresponds to one dataset. Y-axis, the distribution of Spearman's correlation coefficients in 5-fold cross validation tests. Each data point is the correlation coefficient for one single run.

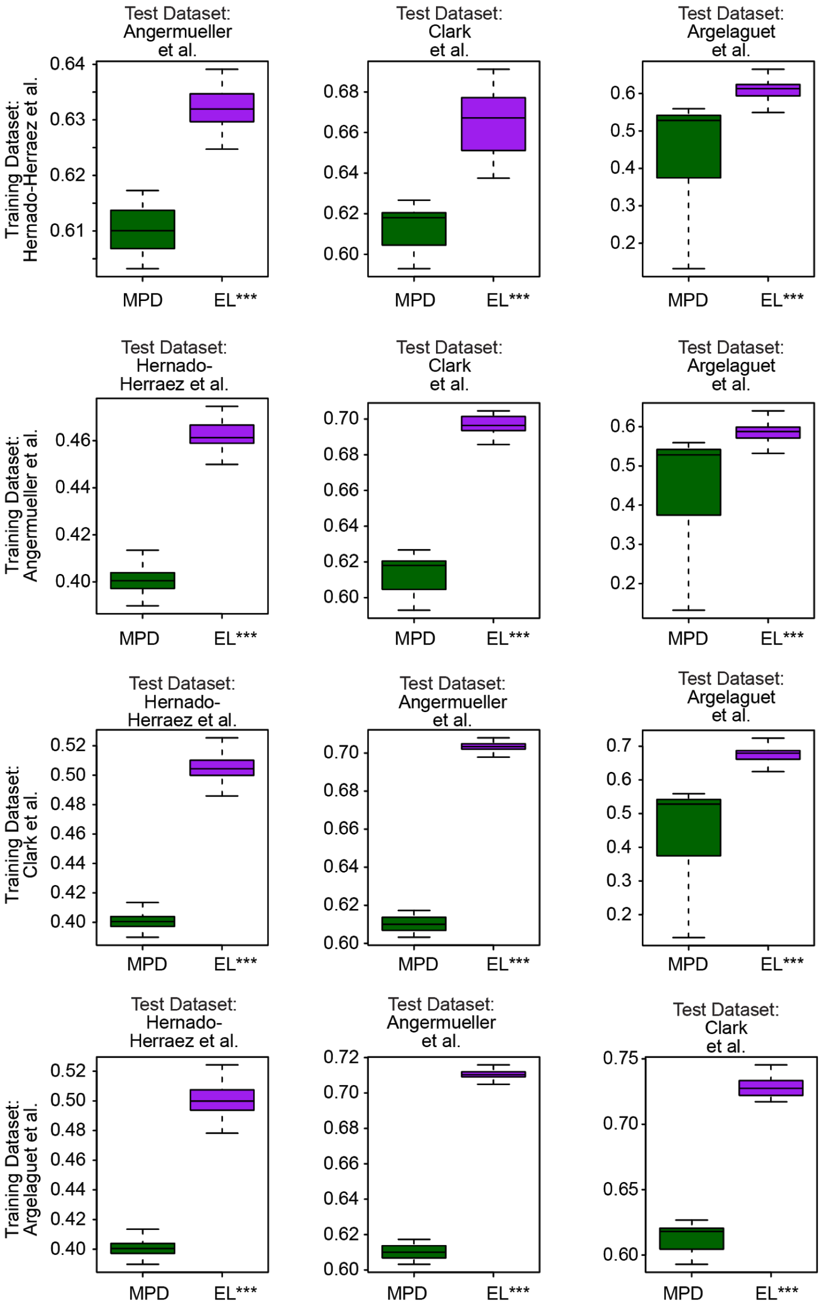

Supplemental Fig. S6. Evaluation of prediction accuracy using Spearman's correlation across genes.

Each row represents the result using the given training dataset and test datasets. Each boxplot shows the distribution of Spearman's correlation coefficients between the predicted gene activity levels and the observed expression levels for the cells in the test dataset. MPD, Mean Promoter De-methylation. EL, Ensemble Learner. Asterisks denote the statistical significance of the difference between MPD and EL (whether EL correlation is statistically higher). ***: one sided *t*-test p-value < 0.001.

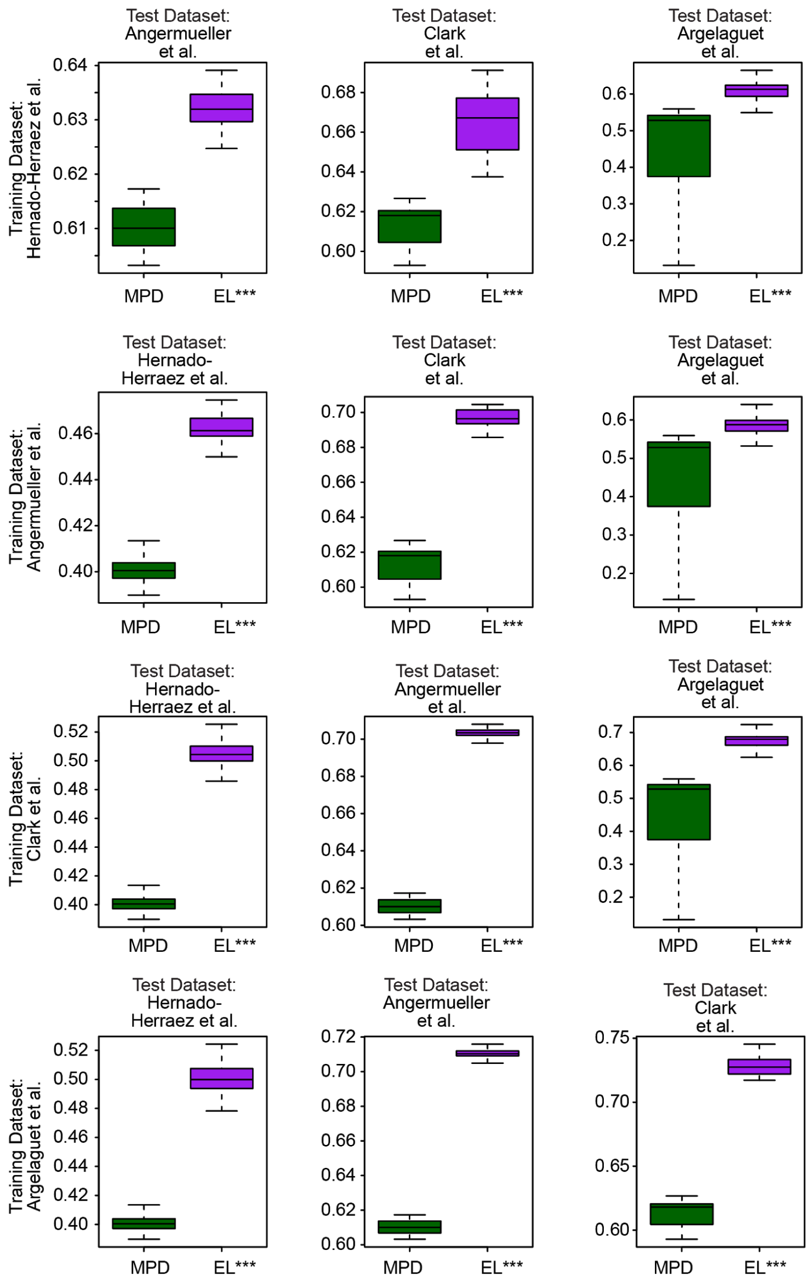

Supplemental Fig. S7. Evaluation of prediction accuracy using median squared error across cells.

Each row represents the result using the given training dataset and test datasets. Each boxplot shows the distribution of the median of the squared difference between the predicted gene activity levels and the observed expression levels for each cell. MPD, Mean Promoter De-methylation, EL, Ensemble Learner. Asterisks denote the statistical significance of the difference between MPD and EL (whether EL error is statistically lower). ***: one sided *t*-test p-value < 0.001.

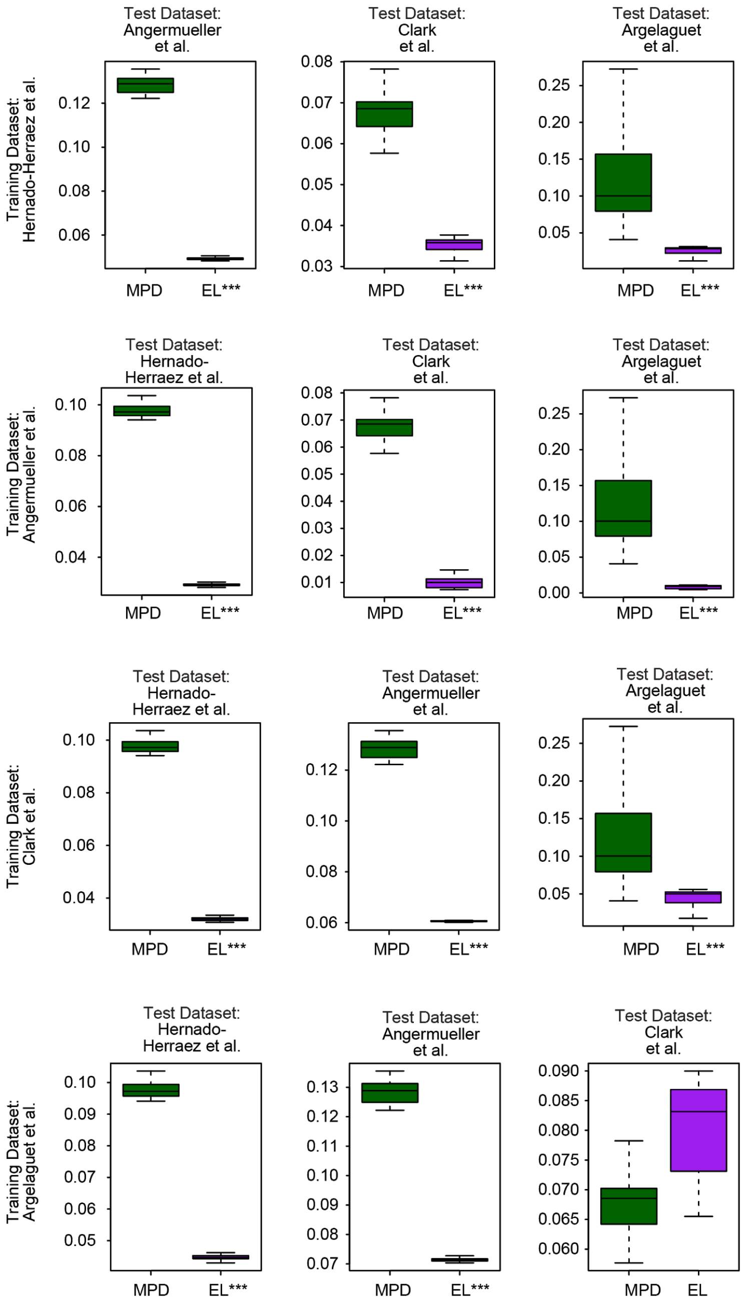

Supplemental Fig. S8. Evaluation of prediction accuracy using median squared error across genes.
Each row represents the result using the given training dataset and test datasets. Each boxplot shows the distribution of the median of the squared difference between the predicted gene activity levels and the observed expression levels for each cell. MPD, Mean Promoter De-methylation, EL, Ensemble Learner. Asterisks denote the statistical significance of the difference between MPD and EL (whether EL error is statistically lower). ***: one sided *t*-test p-value < 0.001.

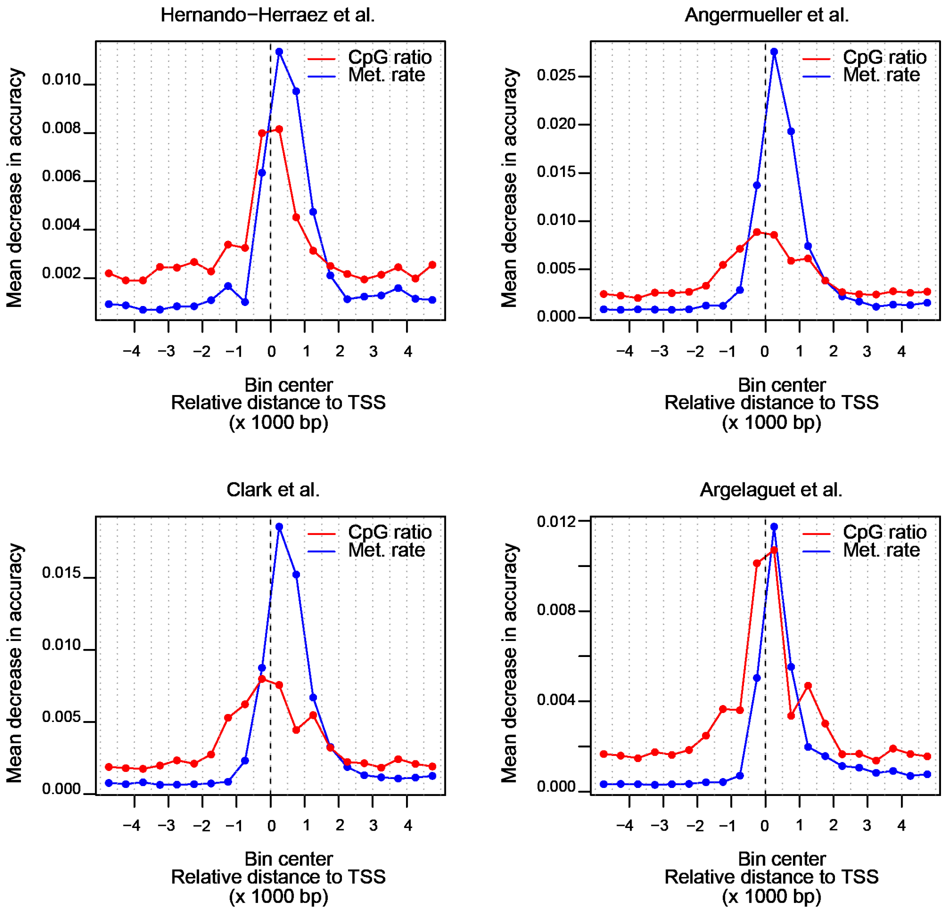

Supplemental Fig. S9. Evaluation of feature importance using Random Forest models.

Each panel corresponds to one training dataset. X-axis, distance from the transcription start site. Gray dotted lines, ends of genomic bins. Y-axis, mean decrease in accuracy of the predictor when values of a feature were permuted. Each colored point corresponds to the center of a bin on the X-axis.

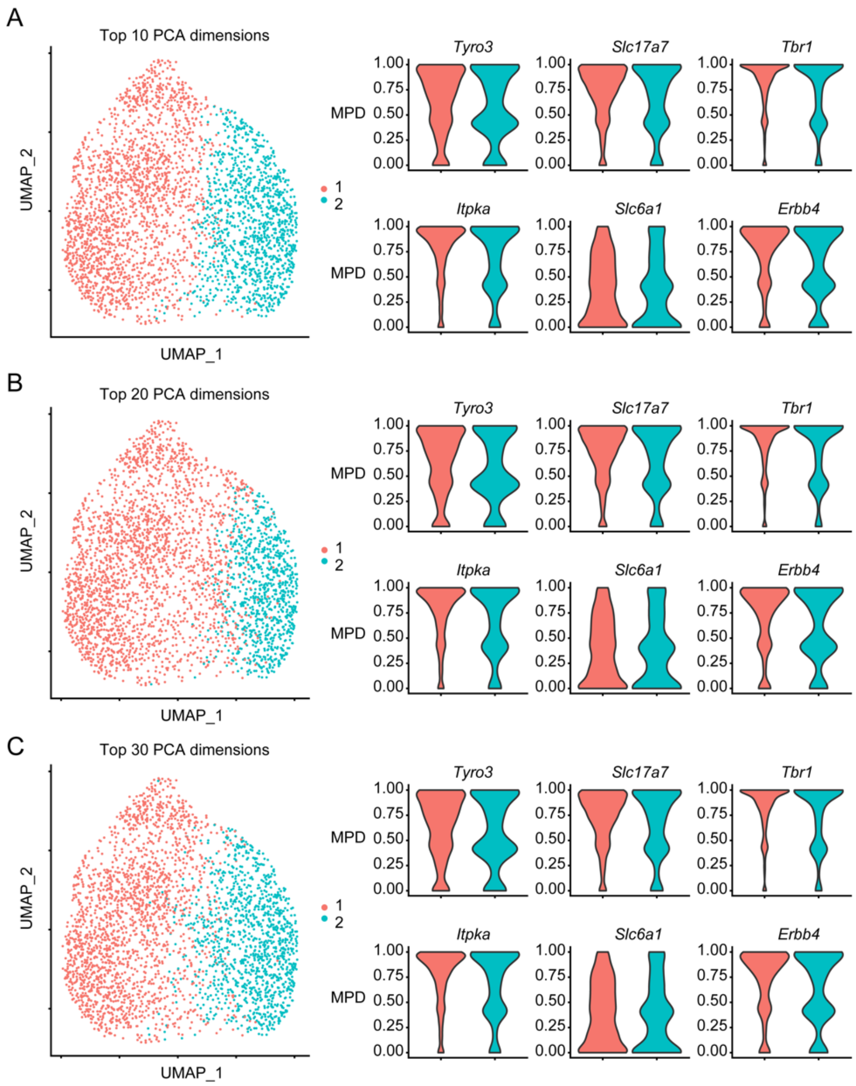

Supplemental Fig. S10. Clustering of neuronal dataset in Fig. 3a using different sets of PCA dimensions with MPD as the input.

A) Clustering with top 10 PCA dimensions (left), expression signature of exhibitory and inhibitory cell markers **B)** Same as panel A, but with top 20 PCA dimensions. **C)** Same as panel A, but with top 30 PCA dimensions.

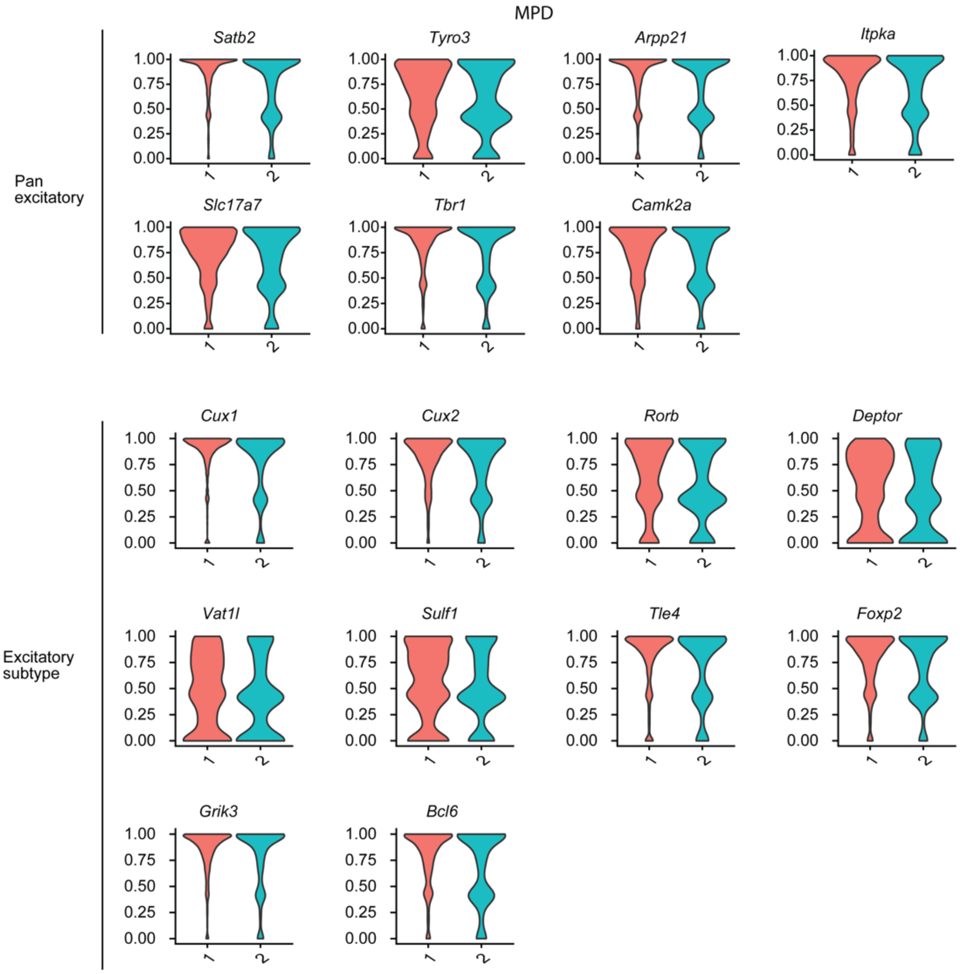

Supplemental Fig. S11. MPD distributions of marker genes for excitatory neurons. X-axis and colors represent different clusters. Y-axis, mean promoter de-methylation (MPD) values for each marker gene.

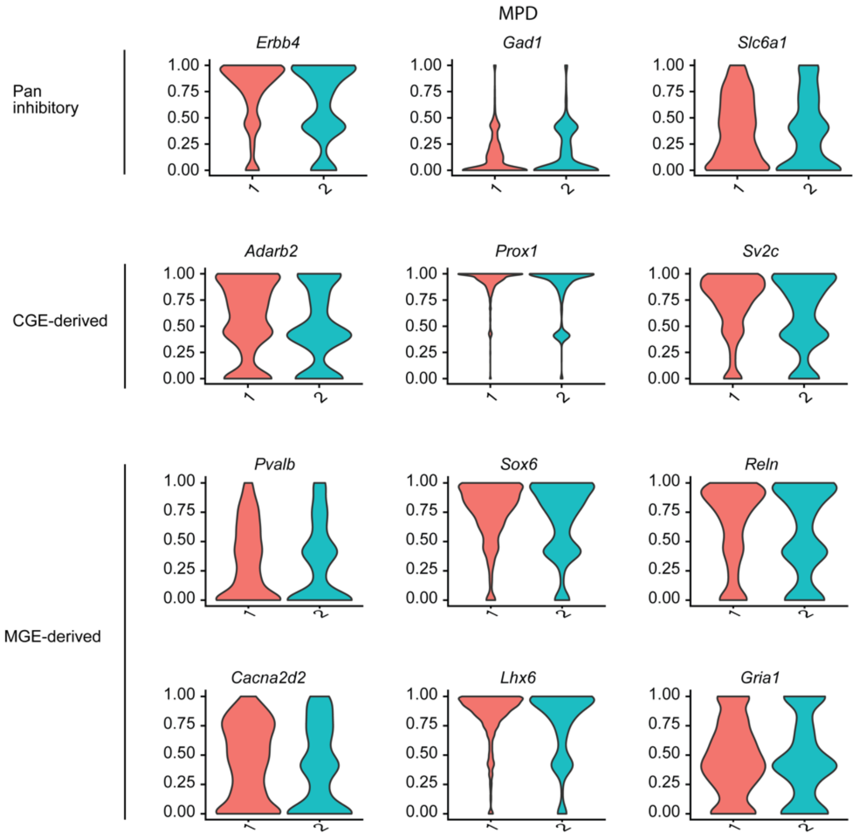

Supplemental Fig. S12. MPD distributions of marker genes for inhibitory neurons. X-axis and colors represent different clusters. Y-axis, mean promoter de-methylation (MPD) values for each marker gene. CGE: Caudal ganglionic eminence. MGE: Medial ganglionic eminence.

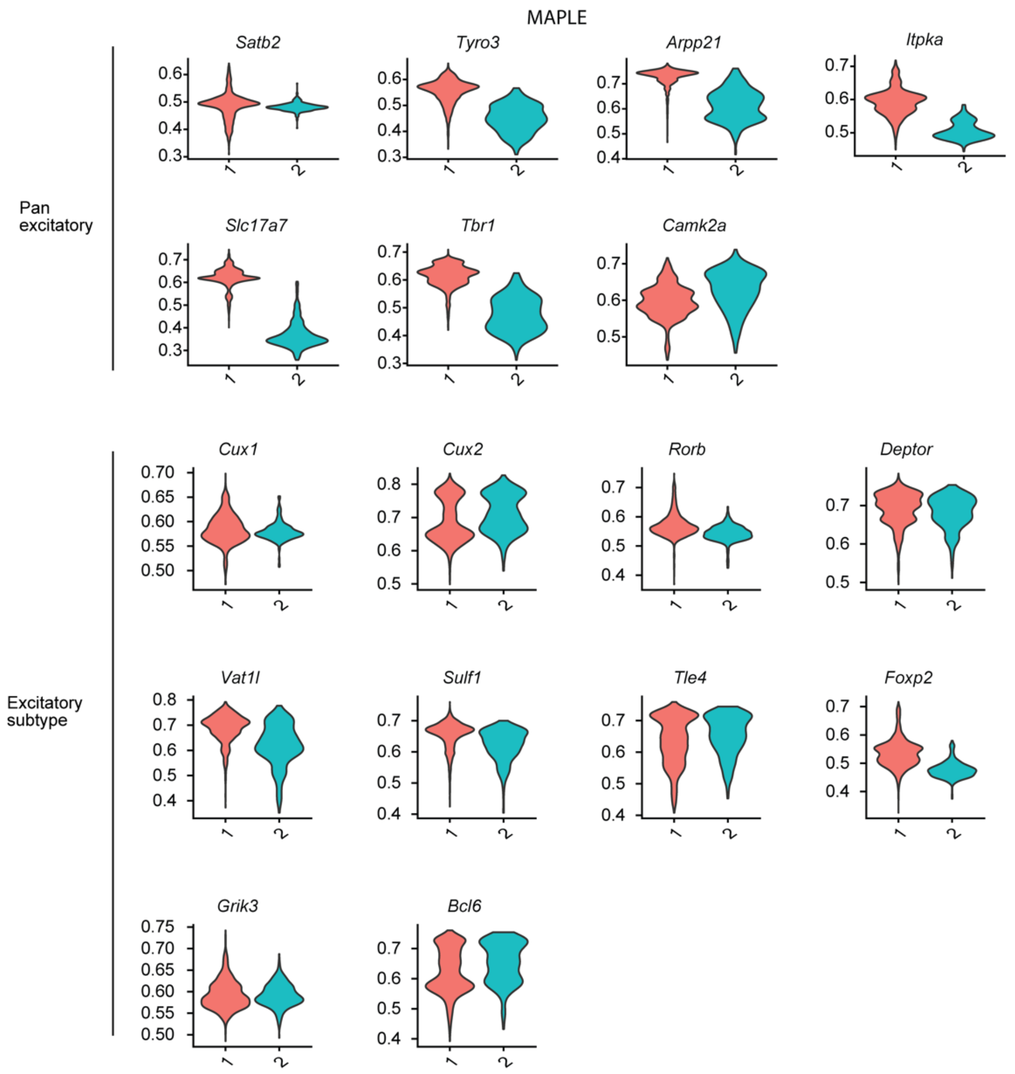

Supplemental Fig. S13. MAPLE gene activity distributions of marker genes for excitatory neurons.

X-axis and colors represent different clusters. Y-axis, MAPLE predicted gene activity values for each marker gene.

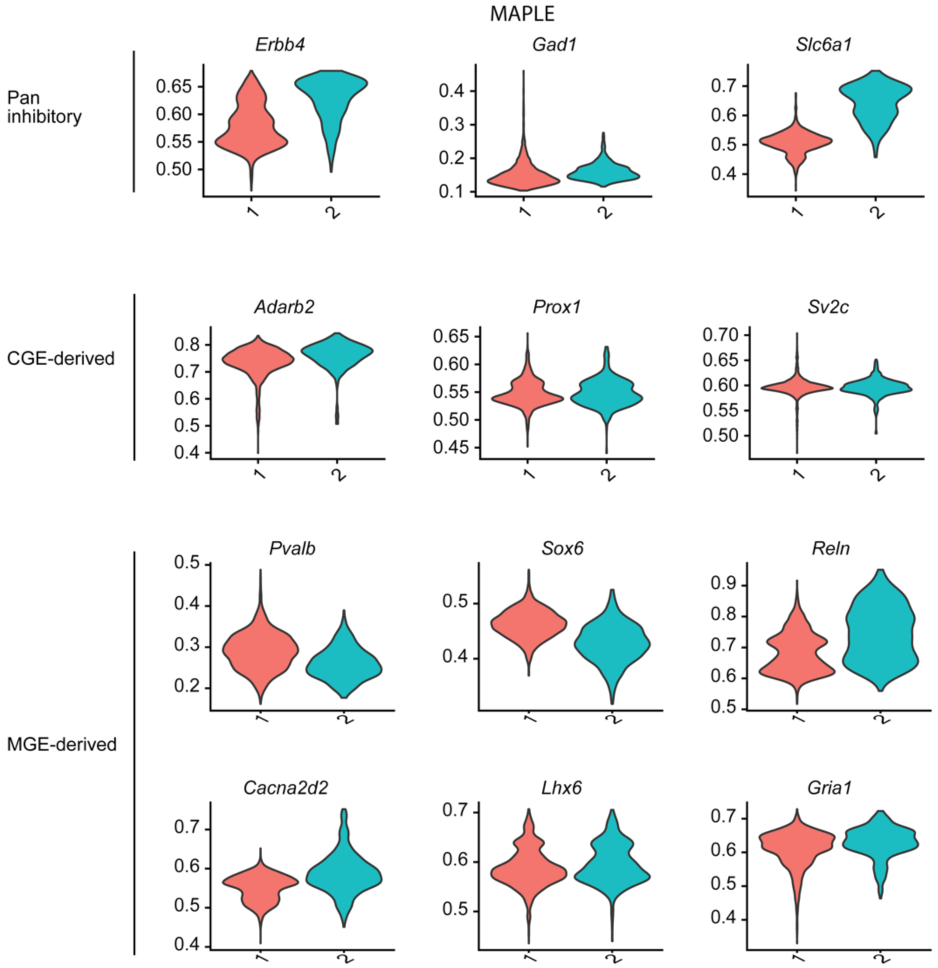

Supplemental Fig. S14. MAPLE predicted gene activity distributions of marker genes for inhibitory neurons.

X-axis and colors represent different clusters. Y-axis, MAPLE predicted gene activity values for each marker gene. CGE: Caudal ganglionic eminence. MGE: Medial ganglionic eminence.

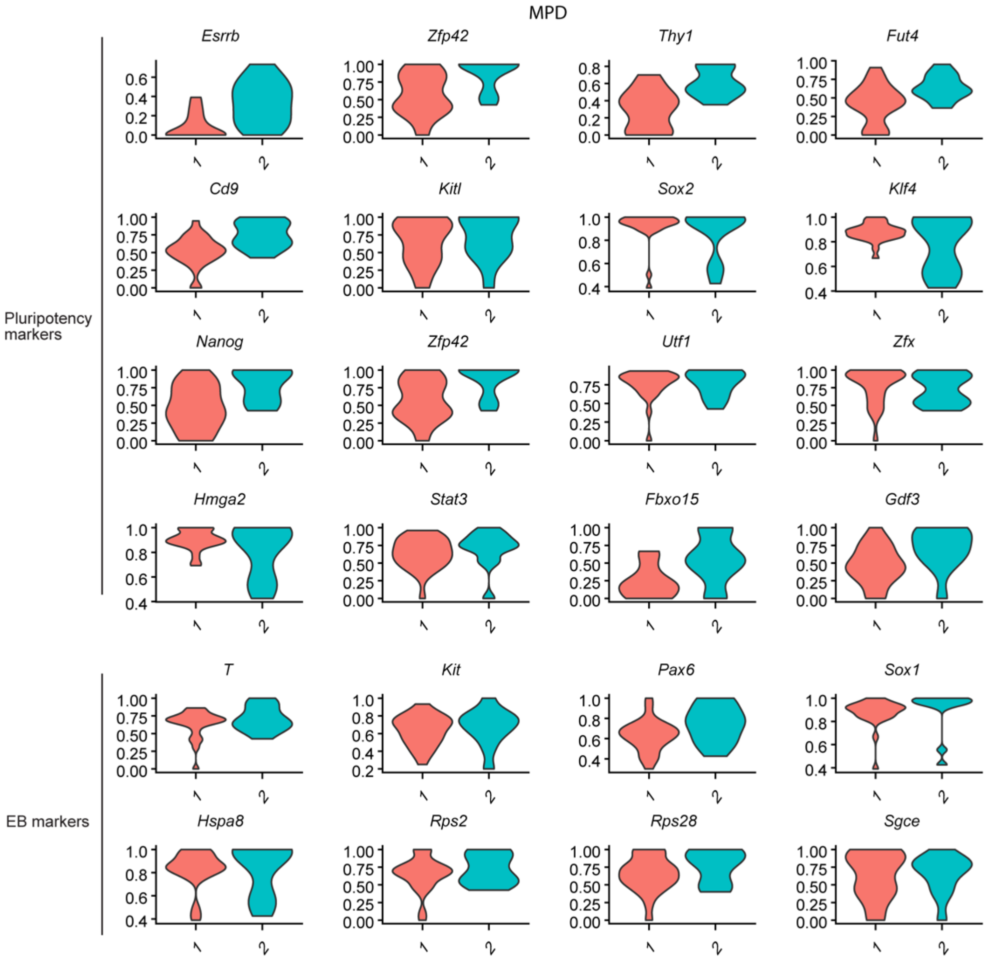

Supplemental Fig. S15. MPD distributions of marker genes for pluripotency and differentiation (EB) in the dataset of Clark et al.

X-axis and colors represent different clusters. Y-axis, MPD values for each marker gene.

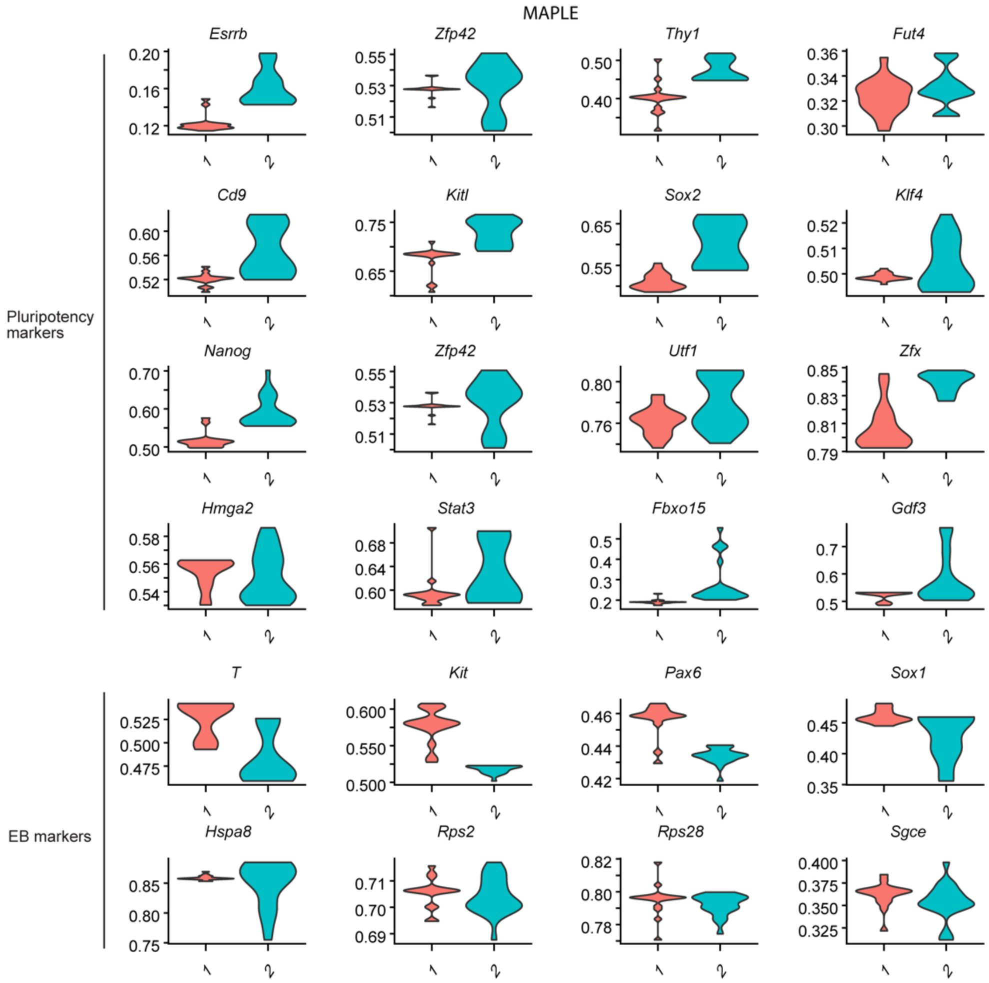

Supplemental Fig. S16. MAPLE predicted gene activity distributions of marker genes for pluripotency and differentiation (EB) in the dataset of Clark et al.
X-axis and colors represent different clusters. Y-axis, MAPLE predicted gene activity values for each marker gene.

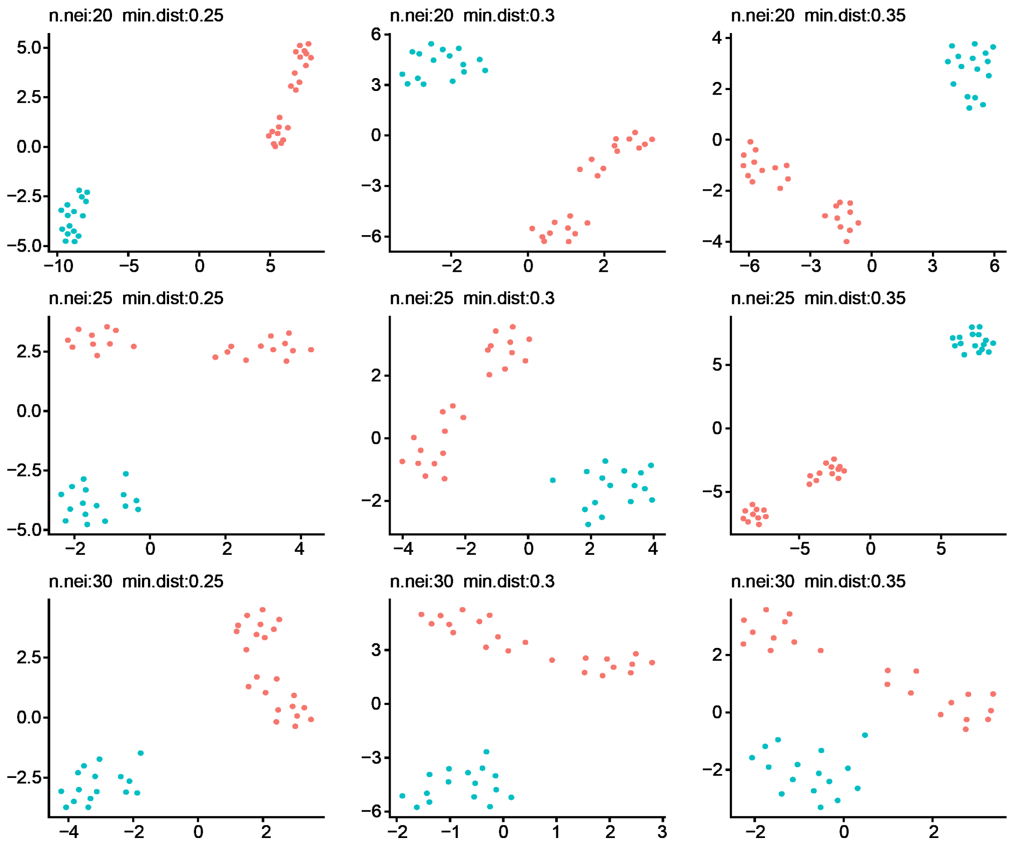

Supplemental Fig. S17. UMAP plots for embryoid bodies (EBs) for the dataset of Clark et al. using various parameter settings.

The parameter settings (n.nei: number of neighbors; min.dist: minimum distance) are shown on the top of each plot. Colors represent different clusters.

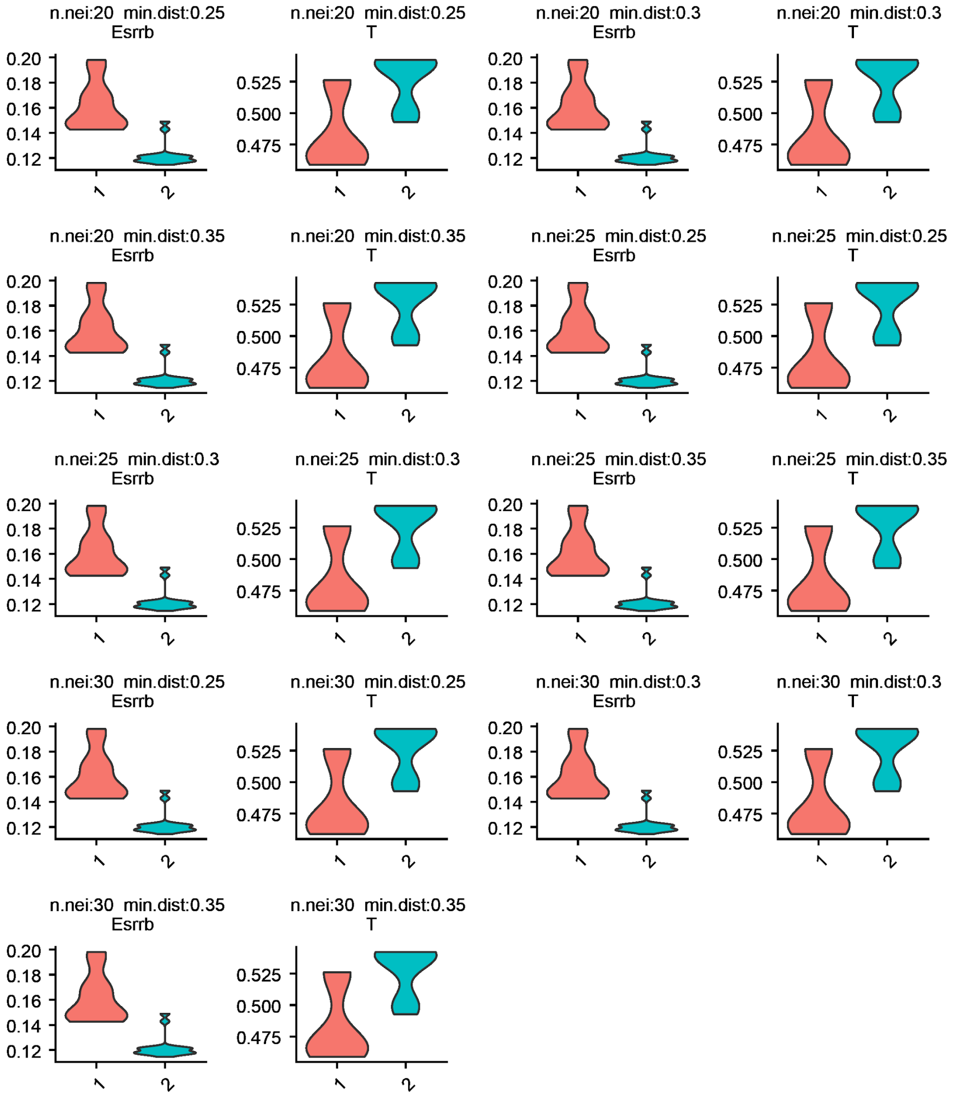

Supplemental Fig. S18. Violin plots showing the MAPLE predicted gene activities for the pluripotent marker gene *Esrrb* and the differentiation marker gene *T* with different parameter settings for embryoid bodies (EBs) for the dataset of Clark et al using various parameter settings.

Colors represent the clusters in Supplemental Fig. S17.

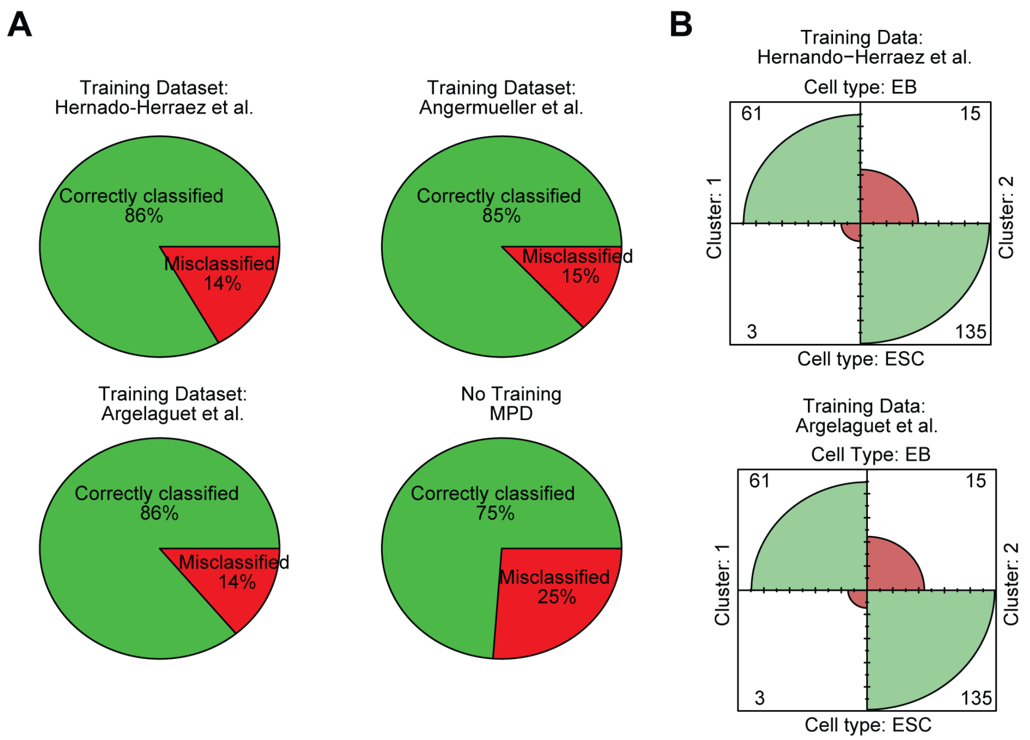

Supplemental Fig. S19. Classification accuracy for cells in the methylome data based on the integration of the dataset of Clark and colleagues (Clark et al. 2018)**.** **A)** Each pie chart shows the percentage of cells that were assigned to the correct cell type (EB or ESC) using k-Nearest Neighbor (kNN) classification based on scRNA data in the integrated PCA. Bottom right chart shows the percentage of correctly classified cells when mean promoter de-methylation was used for the integration. Remaining three panels show the percentage of correct classification with ensemble method using three different training sets. Supervised predictor outperformed the unsupervised mean de-methylation based predictor, regardless of the training dataset. Chi-square test p-value for the comparison of the three classification results with MPD is 0.06. **B)** Confusion matrices for the class assignments of EB and ES cells. Cluster 1 is composed of EBs and Cluster 2 is composed of ESCs. Ensemble method was used for constructing a gene activity matrix from DNA methylation data and then scRNA and gene activity matrices were integrated with Seurat (Stuart et al. 2019). Each confusion matrix is the result of integration using the gene activity matrix predicted by the models trained by the datasets shown at the top. Chi-square test p-value for comparison of the two confusion matrices is 0.03.

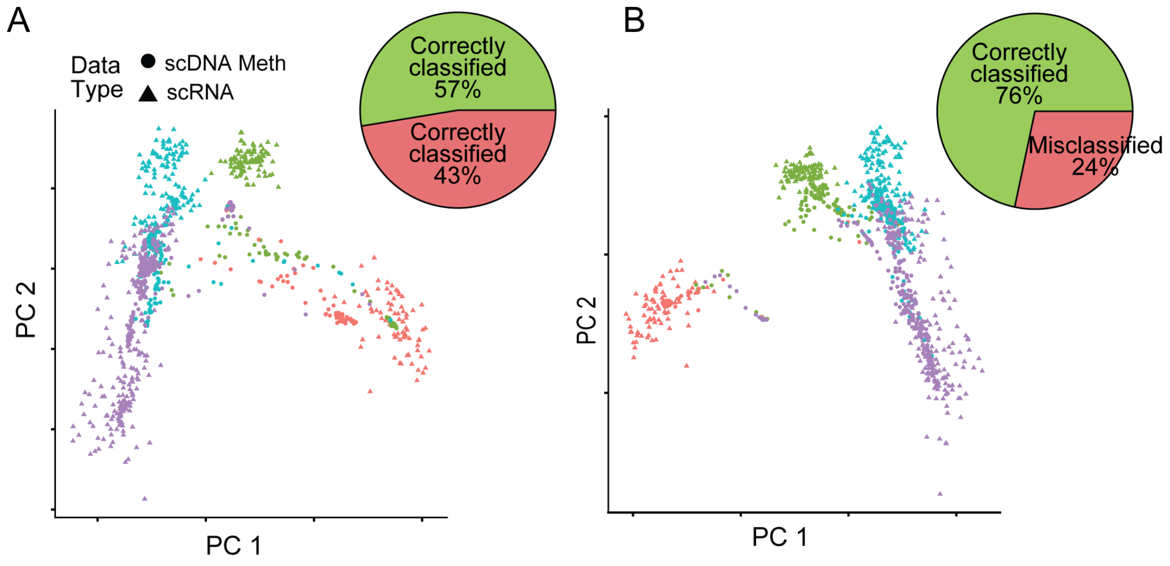

Supplemental Fig. S20. Predictive modeling improves integration with transcriptome data of primary tissues.

A) PCA plots for integrated expression and DNA methylation data. Mean promoter de-methylation (MPD) was used as the input for data integration using Seurat. Pie chart showing the percentage of correctly and mis-classified cells using scDNA-methylation data, based on k-nearest neighbor (kNN) classification on the scRNA-seq cells for the MPD based PCA **B)** Same as panel A), but using MAPLE predicted gene activity as the input.

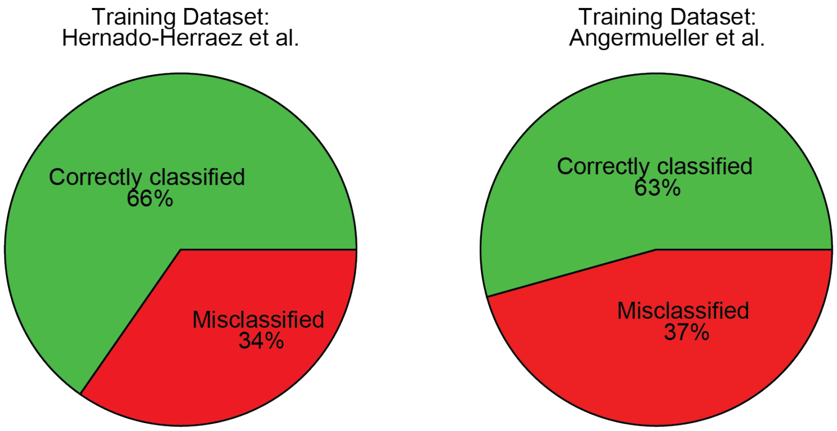

Supplemental Fig. S21. Percentage of correct classification for the cells in the methylome data based on the integration of the dataset of Argelaguet and colleagues (Argelaguet et al. 2019) **using two different training datasets.**
Each pie chart shows the percentage of cells in DNA methylation data that were assigned to the correct cell type (same embryonic day) using k-Nearest Neighbor (kNN) classification using cells from scRNA data in the integrated PCA. The models were trained with the dataset shown on top of each pie chart (Fig. 5d showing the results with the training dataset of Clark and colleagues.). Chi-square test p-values (compared to the pie chart for MPD in Fig. 5c) are 0.04 (left) and 0.07 (right), respectively.

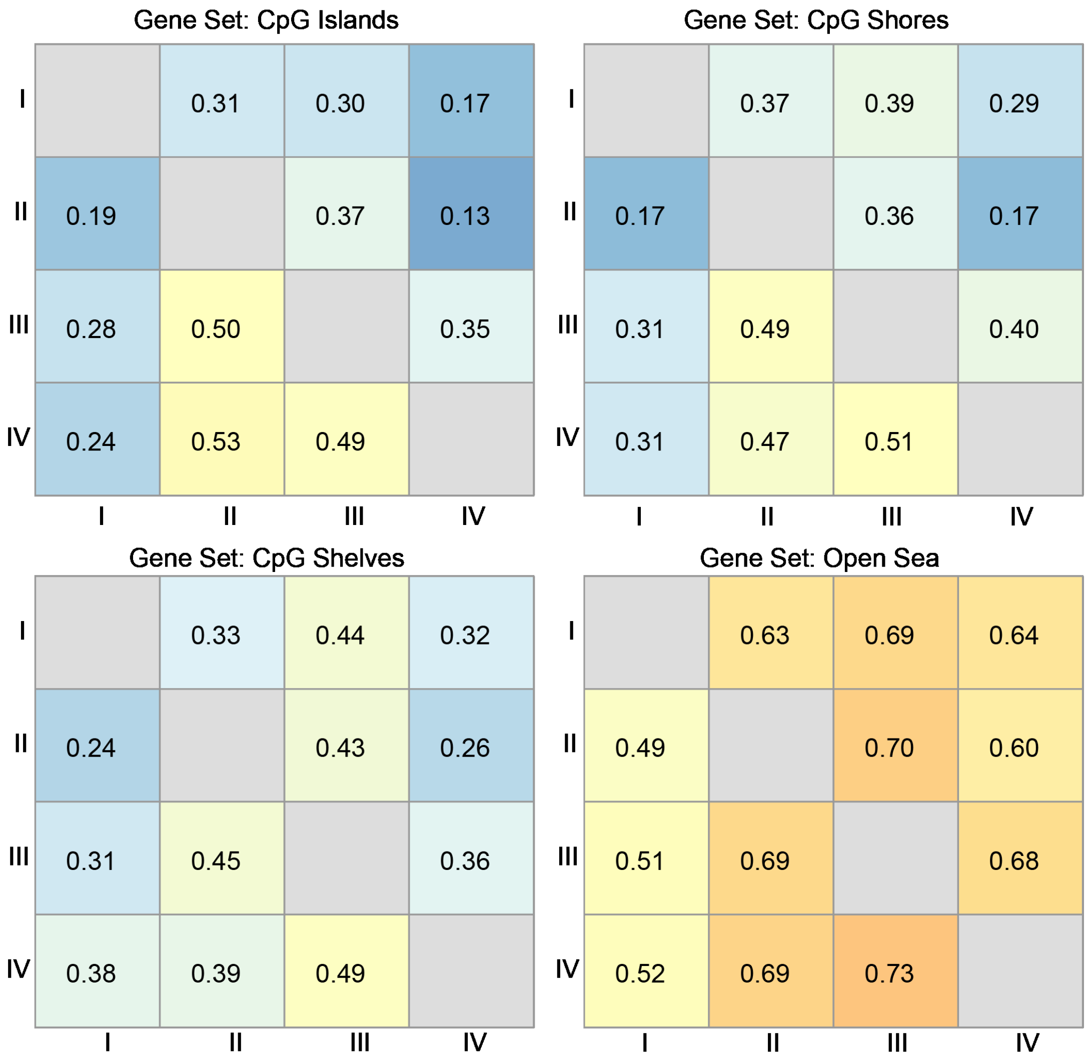

Supplemental Fig. S22. Heatmaps showing Spearman's correlation coefficients between gene expression and MAPLE predicted gene activity for different sets of genes.

Annotation for CpG islands were retrieved from the UCSC Table Browser. CpG shores are +/-2kb flanking regions of CpG islands. CpG shelves are defined as +/-4kb to +/-2kb flanking regions of CpG islands. "Open sea" represents all genes that are not in CpG islands, or shores or shelves. Rows represent the training datasets and columns represent the test datasets. Datasets: I:Hernando-Herraez et al. II: Angermueller et al. III: Clark et al. IV: Argelaguet et al.

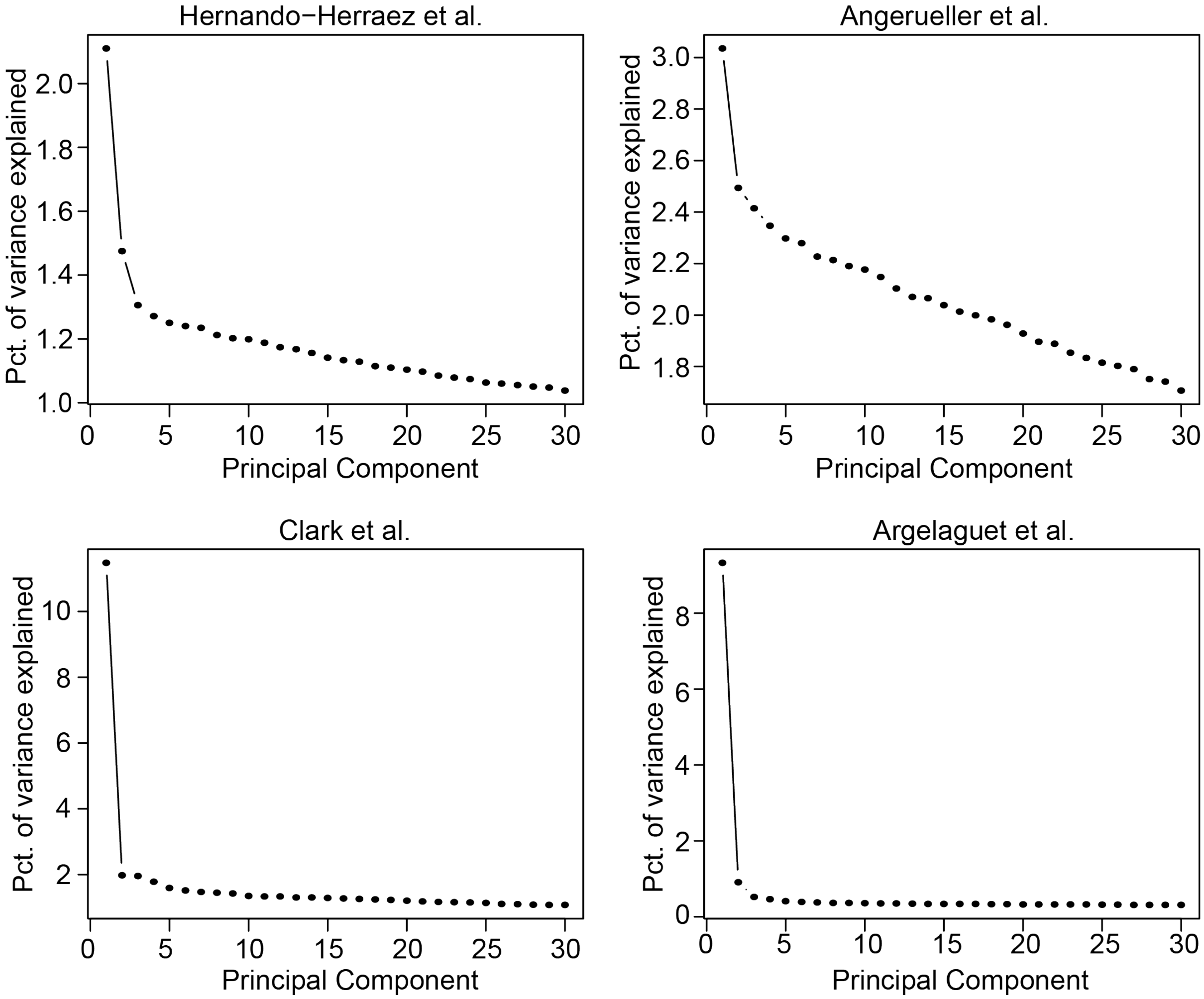

Supplemental Fig. S23. Elbow plots showing the percentage of variance in the data explained by each principal component in the principal component analysis, sorted from highest to lowest.

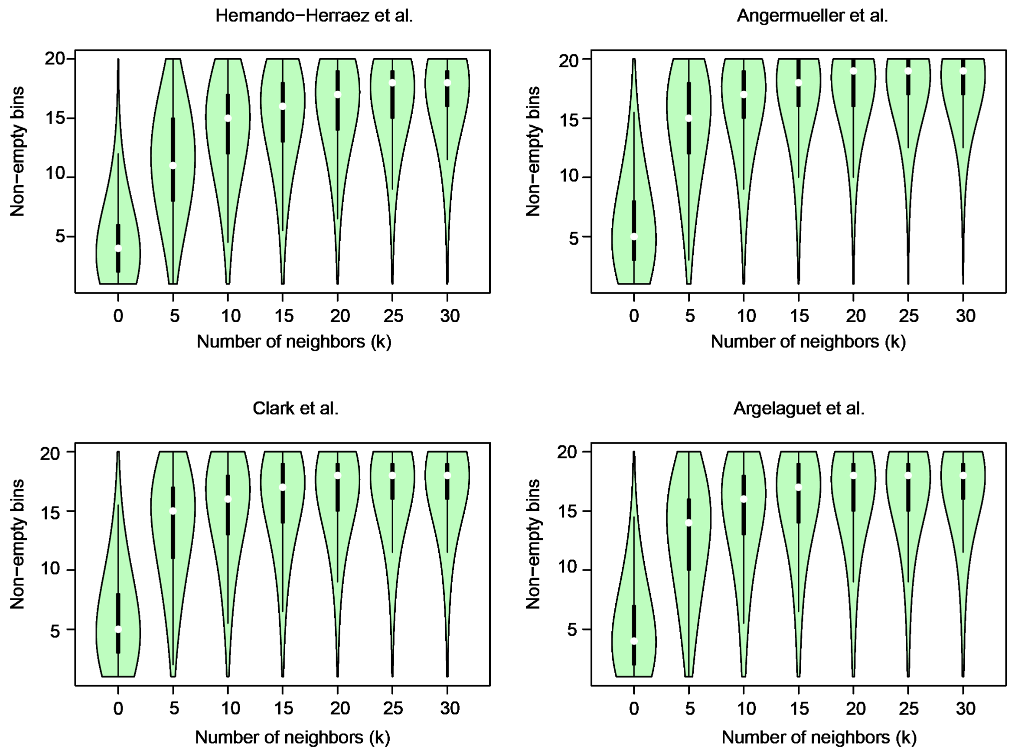

Supplemental Fig. S24. Violin plots showing the distribution of the number of non-empty bins for individual cells and meta-cells of varying sizes.

Each data point is one cell (k=1) or meta-cell (k>1), and the value is the mean number of non-empty bins for the promoters of that cell. White circle shows the median value. Black rectangle shows the upper and lower quartiles. Whisker shows 1.5 times the interquartile range.

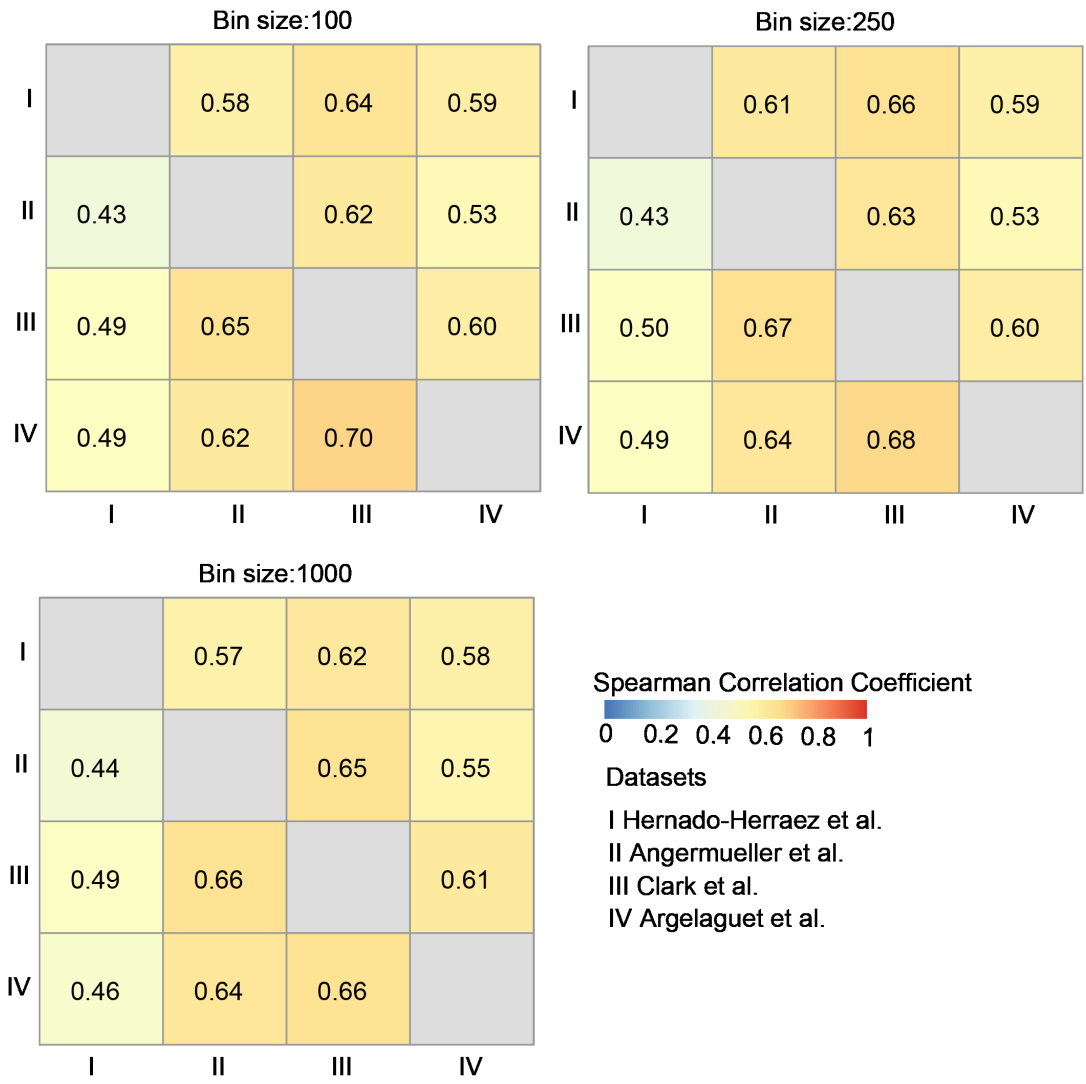

Supplemental Fig. S25. Sensitivity of MAPLE for different bin sizes.
Each heatmap shows the performance of MAPLE when trained and tested with a different bin size. Spearman's correlation coefficients are shown in the cells in the heatmap for different training and test set combinations, rows correspond to the training sets and columns correspond to test sets.

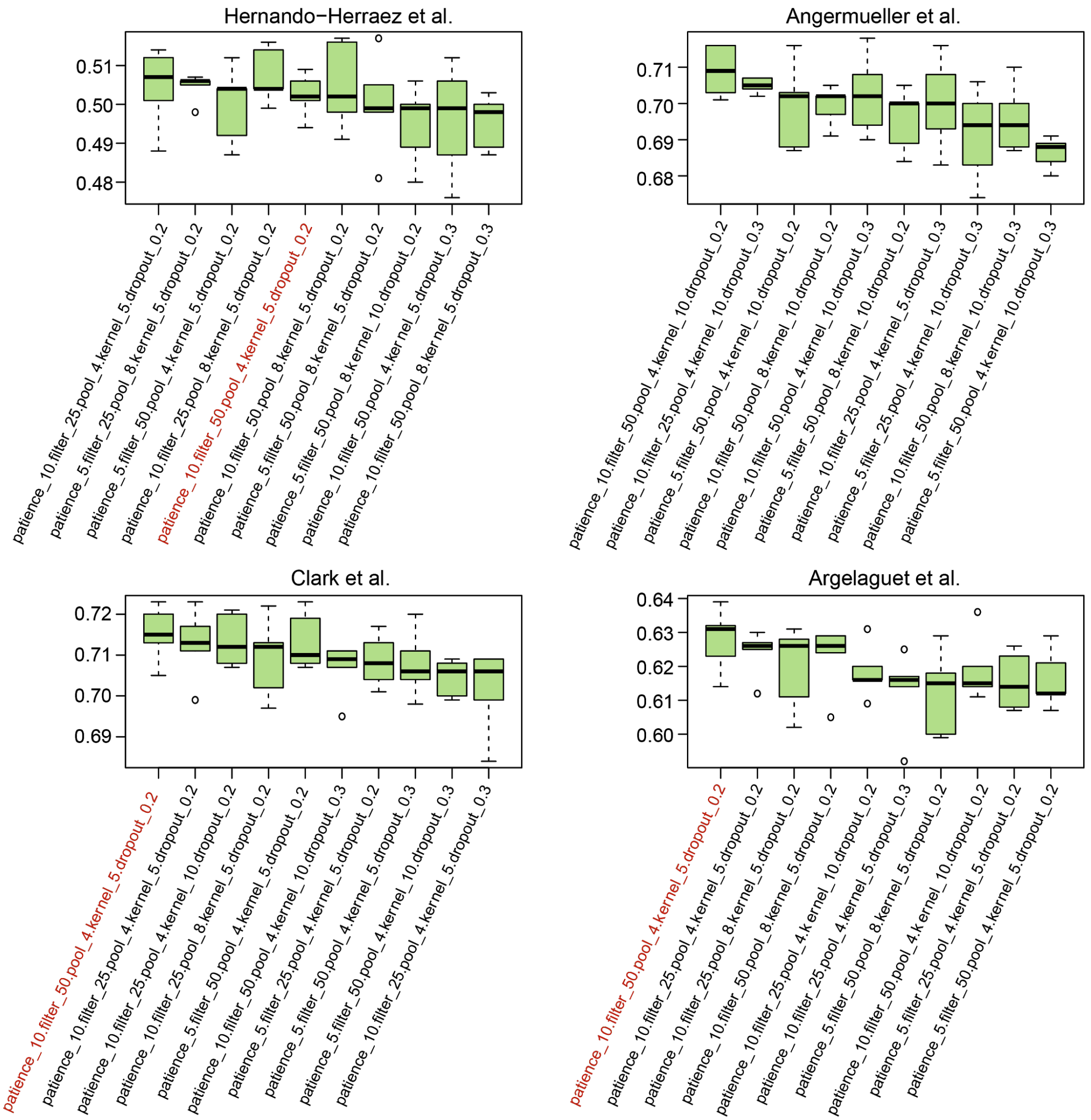

Supplemental Fig. S26. Box plots showing the parameter tuning results with 5-fold cross validation for convolutional neural network predictor.

Top 10 parameter settings with highest Spearman's correlation (median of 5 runs) between the predicted gene activity and observed expression for all the cells and genes are shown. Each data point is a single run. Parameter name is followed by the value for each setting. Patience, number of epochs to stop if there is no reduction in error during optimization step; filter, number of filters; pool, pooling size; kernel, kernel size; dropout, dropout rate. Parameter setting used in the ensemble predictor is highlighted in red.

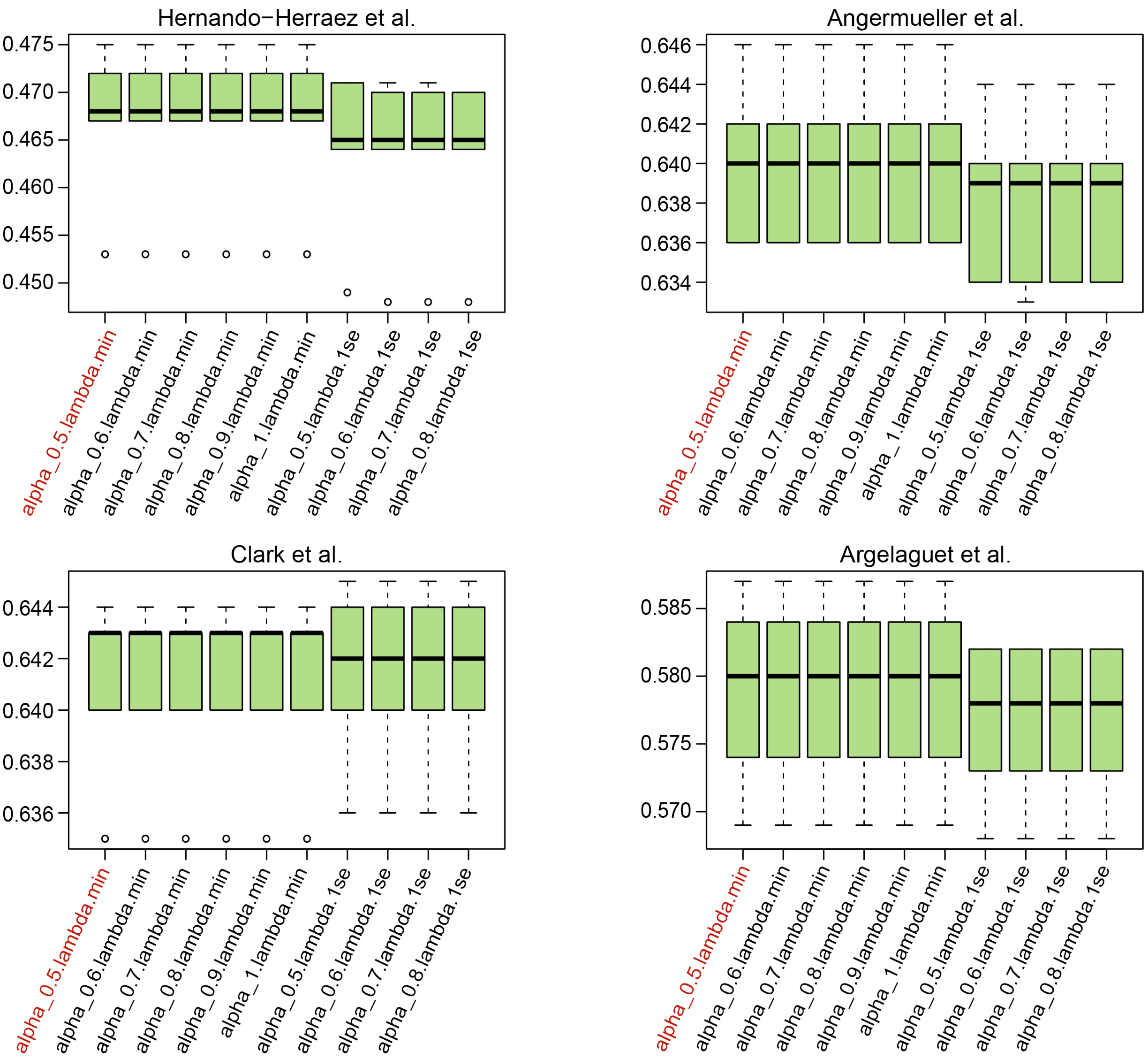

Supplemental Fig. S27. Box plots showing the parameter tuning results with 5-folds cross validation for elastic net predictor.

Top 10 parameter settings with highest correlation are shown. Each data point is a single run. Parameter name is followed by the value for each setting. alpha, alpha value setting the balance between LASSO and Ridge regressions. lambda.min, choice of lambda that gives the minimum error via internal cross validation. lambda-1se, choice of lambda in one standard error vicinity of the lambda.min, for regularization. Parameter setting used in the ensemble predictor is highlighted in red.

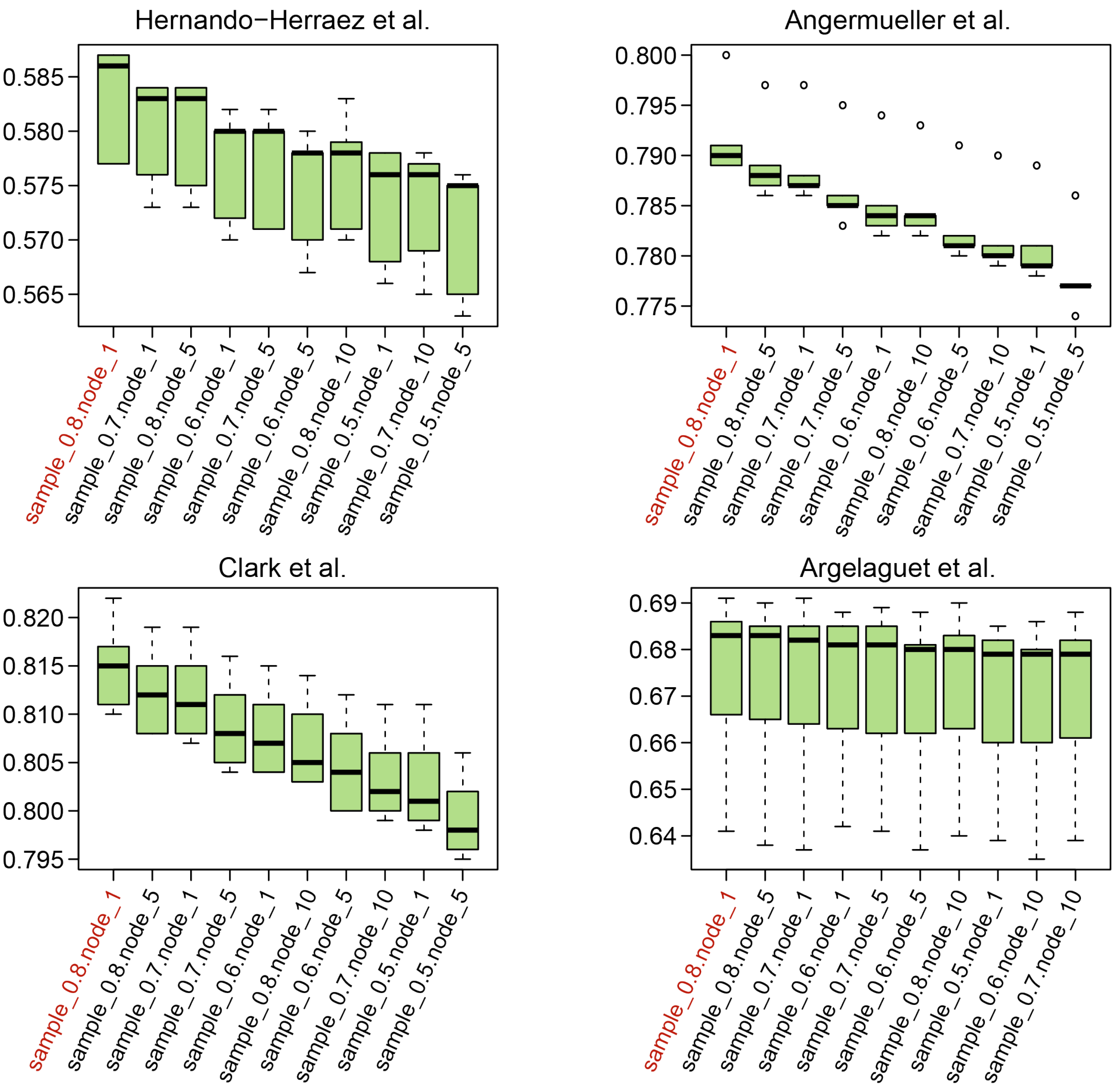

Supplemental Fig. S28. Box plots showing the parameter tuning results with 5-folds cross validation for random forest predictor.

Top 10 parameter settings with highest correlation are shown. Each data point is a single run. Parameter name is followed by the value for each setting. sample, ratio of the input samples to be used for constructing each tree. node, number of minimum terminal nodes. Parameter setting used in the ensemble predictor is highlighted in red.

IV. SUPPLEMENTAL REFERENCES

Angermueller, Christof, Stephen J. Clark, Heather J. Lee, Iain C. Macaulay, Mabel J. Teng, Tim Xiaoming Hu, Felix Krueger, et al. 2016. “Parallel Single-Cell Sequencing Links Transcriptional and Epigenetic Heterogeneity.” *Nature Methods* 13 (3): 229–32.

Argelaguet, Ricard, Stephen J. Clark, Hisham Mohammed, L. Carine Stapel, Christel Krueger, ChantrioInt-Andreas Kapourani, Ivan Imaz-Rosshandler, et al. 2019. “Multi-Omics Profiling of Mouse Gastrulation at Single-Cell Resolution.” *Nature*. https://doi.org/10.1038/s41586-019-1825-8.

Clark, Stephen J., Ricard Argelaguet, ChantrioInt-Andreas Kapourani, Thomas M. Stubbs, Heather J. Lee, Celia Alda-Catalinas, Felix Krueger, et al. 2018. “scNMT-Seq Enables Joint Profiling of Chromatin Accessibility DNA Methylation and Transcription in Single Cells.” *Nature Communications* 9 (1): 781.

Hernando-Herraez, Irene, Brendan Evano, Thomas Stubbs, Pierre-Henri Commere, Marc Jan Bonder, Stephen Clark, Simon Andrews, Shahragim Tajbakhsh, and Wolf Reik. 2019. “Ageing Affects DNA Methylation Drift and Transcriptional Cell-to-Cell Variability in Mouse Muscle Stem Cells.” *Nature Communications* 10 (1): 4361.

Stuart, Tim, Andrew Butler, Paul Hoffman, Christoph Hafemeister, Efthymia Papalexi, William M. Mauck 3rd, Yuhan Hao, Marlon Stoeckius, Peter Smibert, and Rahul Satija. 2019. “Comprehensive Integration of Single-Cell Data.” *Cell* 177 (7): 1888–1902.e21.
